# Global Prevalence of Vancomycin‐Resistant *Enterococcus faecium* and *Enterococcus faecalis* Isolated From Clinical and Environmental Sources: A Systematic Review and Meta‐Analysis

**DOI:** 10.1002/mbo3.70434

**Published:** 2026-10-13

**Authors:** Negar Narimisa, Tahereh Navidifar, Tuqa T. Hameed, Roghayeh Mohammadzadeh, Faranak Abdoli, Mahsa Ghamari, Delaram Nourollahi, Fatemeh Ansari, Arefeh Ansari, Narjess Bostanghadiri

**Affiliations:** ^1^ Department of Microbiology, School of Medicine Iran University of Medical Sciences Tehran Iran; ^2^ Department of Basic Sciences Shoushtar Faculty of Medical Sciences Shoushtar Iran; ^3^ College of Dentistry University of Al Maarif Al Anbar Iraq; ^4^ Food Safety Research Center (salt) Semnan University of Medical Sciences Semnan Iran; ^5^ Research Center for Clinical Virology Tehran University of Medical Sciences Tehran Iran; ^6^ Department of Pathobiology, School of Public Health Tehran University of Medical Sciences Tehran Iran; ^7^ Science at Capilano University

**Keywords:** *E. faecalis*, *E. faecium*, global prevalence, non‐clinical sources, vancomycin‐resistant enterococci, VRE

## Abstract

Vancomycin‐resistant enterococci (VRE) are an increasing public health concern. However, the contribution of environmental reservoirs to the global epidemiology of vancomycin resistance remains unclear. This meta‐analysis estimated the prevalence of Vancomycin‐resistant *Enterococcus faecalis* (VREfs) and vancomycin‐resistant *Enterococcus faecium* (VREfm) from clinical and environmental specimens worldwide. We conducted a systematic literature search in PubMed, Web of Science, Embase, and Scopus up to 26 April 2025. Statistical analysis was performed using the meta and metaphor packages in R, and heterogeneity was evaluated using I^2^. We assessed potential publication bias using Egger's test. A total of 349 eligible studies were included. The pooled prevalence of VREfm was 25%, and VREfs was 5%. The prevalence of both VREfs and VREfm increased significantly during the last two decades. The highest prevalence of VREfm and VREfs was found in rectal swabs (76%), hospital environmental samples (74%), environmental water (26%), and animal stool (40%). By integrating clinical and environmental data, the present study highlights the possible role of non‐clinical reservoirs in the persistence and dissemination of VRE. Our results support the One Health approach for VRE surveillance and emphasize the need for environmental surveillance in infection prevention and antimicrobial resistance control strategies.

## Graphical abstract

A global systematic review and meta‐analysis of 375 studies found increasing VRE prevalence over the past two decades, with higher prevalence of VRE *E. faecium* than *E. faecalis*. High prevalence in clinical and environmental reservoirs highlights the importance of integrated One Health surveillance to limit VRE dissemination.

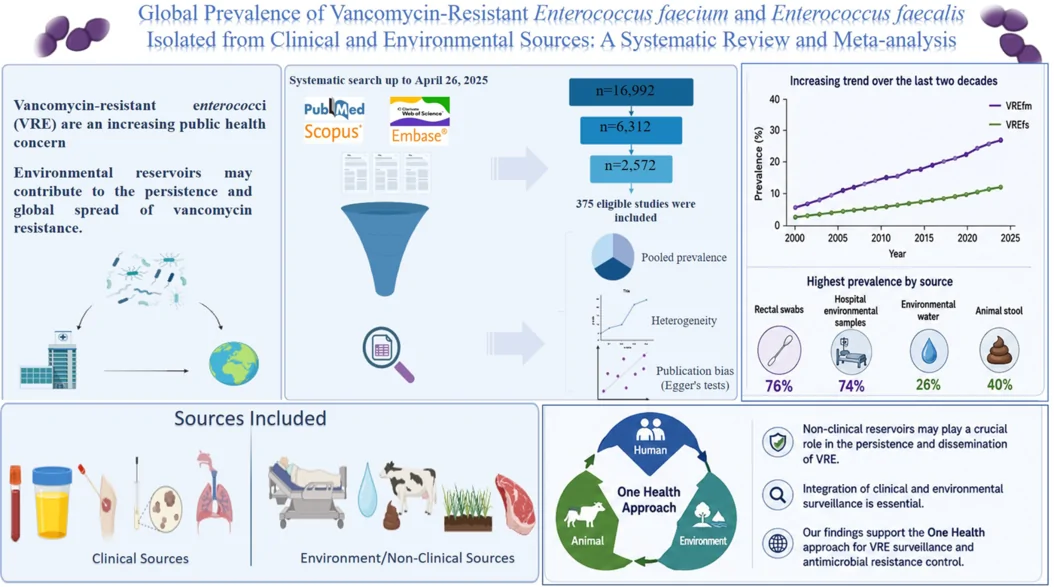

## 1 Introduction

*Enterococcus* species are a group of Gram‐positive, facultatively anaerobic, and extremely tolerant bacteria. They are often normal colonizers of the human gastrointestinal tract and can also be isolated from other organs as part of the microbiota, including the skin, genitourinary tract, and oral cavity. *Enterococcus* spp. can act as opportunistic pathogens and cause serious infections such as bloodstream infections, endocarditis, and wound infections. *Enterococcus* spp. are highly adaptable and can survive in unfavorable conditions such as high temperature, alkaline pH, hyperosmotic stress, and high concentrations of bile salts (García‐Solache and Rice 2019). These properties allow them to colonize and survive in the hospital environment and on medical devices such as catheters and ventilators. The extensive use of antibiotics in hospitals increases the chance of emergence and spread of multidrug‐resistant *Enterococci*, one of the most frequent causes of nosocomial infections, especially in patients hospitalized in the ICU (Bhatti et al. 2023a).

*Enterococcus faecalis* and *Enterococcus faecium* are the two clinically relevant species responsible for most human enterococcal infections. These two species are also the fourth most common cause of nosocomial infections (García‐Solache and Rice 2019; Miller and Arias 2024). In particular, *E. faecium* is classified as one of the ESKAPE pathogens because of its high ability to acquire the determinants of multidrug resistance and persist in healthcare settings (Miller and Arias 2024).

Regardless of the *Enterococcus* spp. the high levels of antimicrobial resistance in enterococcal infections limit the available antibiotic treatments (Zaheer et al. 2020). There are major concerns about the ability of *Enterococcus* spp. to develop high‐level resistance to the glycopeptides (such as vancomycin), aminoglycosides, and oxazolidinones (such as linezolid) (Kankalil George et al. 2021; Dadashi et al. 2021).

Vancomycin resistance is mediated by the *van* genes that alter the peptidoglycan synthesis pathway. These genes may be encoded on chromosomes or on mobile genetic elements, which have resulted in the dissemination of vancomycin resistance among *Enterococcus* species (von Wintersdorff et al. 2026; Faron et al. 2016; Depardieu and Courvalin 2017). Vancomycin‐resistant *Enterococcus faecalis* (VREfs) and vancomycin‐resistant *Enterococcus faecium* (VREfm) contribute to increased mortality associated with various infections, especially bacteremia, with E. *faecium* accounting for approximately 77% of cases and *E. faecalis* for 9% (Depardieu and Courvalin 2017).

The epidemiology of Vancomycin‐resistant *Enterococcus* (VRE) isolated from clinical and environmental sources has been studied extensively (Zaheer et al. 2020; von Wintersdorff et al. 2026; Arias and Murray 2012; Bhatti et al. 2023b; Monticelli et al. 2021; Rice et al. 1995; Staley et al. 2014). These studies have shown that the increasing prevalence of VRE reduces the effectiveness of glycopeptide therapy and highlights the need for ongoing epidemiological surveillance and monitoring of vancomycin resistance patterns. This therapeutic limitation has increased reliance on last‐resort agents such as linezolid, daptomycin, and tigecycline. Linezolid and daptomycin are important options for treatment of serious VRE infections, notably bacteremia. However, comparative clinical data are limited, and therapy choice may be influenced by antibiotic resistance, site of infection, and patient factors. In some infections, tigecycline may be used as an alternative, but its efficacy for serious VRE infections such as bacteremia and endocarditis is not fully established (Cairns et al. 2023).

The majority of previous systematic review and meta‐analysis studies have focused on clinical VRE isolates from individual geographic regions (Emaneini et al. 2016; Huang et al. 2025; Jabbari Shiadeh et al. 2019; Shrestha et al. 2021; Smout et al. 2023). However, only a limited number of meta‐analyses have investigated integrated data from both clinical and environmental VRE isolates at the national or continental level (Alemayehu and Hailemariam 2020; Wada et al. 2020). Also, a global One Health meta‐analysis recently investigated the VRE prevalence across human, livestock, and food‐animal populations; however, environmental sources such as water and healthcare‐associated environments were not comprehensively incorporated (Caddey et al. 2025). Nevertheless, no previous global systematic review and meta‐analysis has comprehensively integrated data from both clinical and environmental isolates of *E. faecium* and *E. faecalis* at a global level within a single analytical approach.

Moreover, environmental *Enterococcus* spp. may serve as potential reservoirs of vancomycin resistance determinants and contribute to the transmission of resistance genes to hospital‐associated enterococcal populations (Zaheer et al. 2020). Hence, simultaneous evaluation of clinical and environmental sources is essential for an in‐depth understanding of the epidemiology of VRE from a One Health perspective.

Unlike previous reviews, the present systematic review and meta‐analysis provides (i) the estimation of global prevalence of vancomycin resistance in *E. faecium* and *E. faecalis* isolated from both clinical and environmental sources; (ii) the distribution of vancomycin resistance among these two species based on geographic regions and different sources, including various clinical and environmental origins; (iii) the evaluation of temporal trends in antimicrobial resistance; and (iv) the assessment of the influence of antimicrobial susceptibility testing (AST) methods and interpretive guidelines on reported vancomycin resistance rates. By addressing these objectives, this study aimed to overcome limitations in the current literature and establish an in‐depth knowledge of trends in vancomycin resistance in *E*. *faecium* and *E. faecalis* globally, considering variations across clinical and environmental sources.

Moreover, species‐specific, region‐specific, and source‐specific resistance patterns could influence risk stratification, optimization of infection prevention and control measures, prioritization of antimicrobial stewardship interventions, and targeted surveillance in healthcare settings. Therefore, a thorough understanding of VRE circulation among clinical and environmental reservoirs may help develop integrated surveillance programs and evidence‐based policies to restrict VRE dissemination and preserve the efficacy of remaining therapeutic options for severe enterococcal infections.

## 2 Methods

### 2.1 Search Strategy and Study Selection

To identify relevant articles, we systematically searched MEDLINE (via PubMed), Embase, Scopus, and Web of Science from database inception to 26 April 2025. We developed the search strategy using combinations of terms related to prevalence or frequency, Enterococcus and its relevant species or synonyms, and vancomycin resistance, with database‐specific adaptations to account for differences in indexing systems and search syntax. In PubMed, the search combined relevant MeSH terms with free‐text terms searched in the Title/Abstract (TIAB) fields. In Embase, free‐text terms were searched within the title and abstract fields (ti, ab). In Scopus, we used the TITLE‐ABS‐KEY field, which covers titles, abstracts, and keywords; in Web of Science, we searched the Topic (TS) field. No restrictions on publication date or language were applied. The complete database‐specific search strategies, including all search terms, Boolean operators, truncation symbols, and field specifications, are provided in Supporting File 1. This systematic review and meta‐analysis was conducted and reported in accordance with the Preferred Reporting Items for Systematic Reviews and Meta‐Analyses (PRISMA) guidelines (Page et al. 2021). The review protocol was prospectively registered in the PROSPERO database (CRD420251082821), supporting transparency and methodological rigor. We merged records retrieved from the databases and removed duplicates using EndNote 20 (Clarivate Analytics, Philadelphia, PA, USA). We then imported the deduplicated records into Rayyan for screening and study selection. Two reviewers independently screened the titles and abstracts of all retrieved records and subsequently assessed the full texts of potentially eligible studies against the predefined eligibility criteria. Reviewers resolved disagreements through discussion, and a third reviewer adjudicated unresolved discrepancies.

### 2.2 Selection Criteria and Data Extraction

Studies were considered eligible if they met the following inclusion criteria: (i) original observational studies (cross‐sectional, cohort, or surveillance studies); (ii) studies reporting the prevalence of VREfm and/or VREfm isolated from clinical or non‐clinical (environmental or animal) sources; (iii) studies providing sufficient data to calculate the prevalence of vancomycin resistance, including the number of VREfs and VREfm isolates and the total number of *E. faecium* or *E. faecalis* isolates examined; and (iv) studies published in peer‐reviewed journals.

Studies were excluded if they were review articles, conference abstracts, editorials, letters, case reports, guidelines, duplicate publications, studies with insufficient data for prevalence estimation, studies with unclear isolate sources or species identification, or studies whose full texts were unavailable despite reasonable efforts to obtain them, including contacting the corresponding authors via email.

Two reviewers independently extracted the data using a standardized data extraction form. We resolved disagreements through discussion and, when necessary, consultation with a third reviewer. The following information was extracted from each eligible study: first author's name, year of publication, country and continent, study period (year of sample collection), study design, source of isolates (clinical or non‐clinical), specimen type (e.g., blood, urine, rectal swab, wound, environmental water, hospital environment, and animal stool), infection setting (hospital‐acquired or community‐acquired), hospital ward (when available), disease or clinical condition, total number of *E. faecium* and/or *E. faecalis* isolates, number of VREfs and VREfm isolates, bacterial identification method, AST method, interpretive guideline used for vancomycin susceptibility testing (e.g., CLSI or EUCAST), and other variables relevant to the predefined subgroup analyses.

### 2.3 Quality Assessment

The methodological quality of the included studies was independently assessed by two reviewers using the Joanna Briggs Institute (JBI) Critical Appraisal Checklist for Studies Reporting Prevalence Data (Supporting Table S1/S2) (Migliavaca et al. 2020). Each study was evaluated across nine methodological domains, with each item scored as “Yes,” “No,” “Unclear,” or “Not Applicable” in accordance with the JBI recommendations. Reviewers resolved disagreements through discussion and consensus. When consensus could not be achieved, a third reviewer independently assessed the study and made the final decision. Based on the total JBI score, studies were categorized as low quality (0–4 points), moderate quality (5–6 points), or high quality (7–9 points).

### 2.4 Statistical Analysis

Data were analyzed using the meta package in R software (version 4.4.3). We estimated the pooled prevalence of VREfs and VREfm using a random‐effects model with the inverse‐variance method. To stabilize the variance of observed proportions, we applied the Freeman–Tukey double arcsine transformation and reported pooled estimates with corresponding 95% confidence intervals (CIs). Given the presence of studies with zero events, we employed the Freeman–Tukey double arcsine transformation. This method provides more reliable weighting of individual studies in the random‑effects model and yields robust pooled estimates on the original scale through appropriate back‑transformation (Abdulmajeed et al. 2025).

Between‐study heterogeneity was assessed using the *I* ^2^ statistic and interpreted as low (*I* ^2^ ≤ 25%), moderate (25% < *I* ^2^ ≤ 75%), or high (*I* ^2^ > 75%). To investigate potential sources of heterogeneity, subgroup analyses were conducted according to year of sample collection, country, continent, infection setting (hospital‐acquired vs. community‐acquired), hospital ward, isolate origin, source of isolates (clinical vs. non‐clinical), disease type, *Enterococcus* diagnostic method, AST method used for VRE detection, interpretive guideline applied, and study quality assessed using the Joanna Briggs Institute (JBI) Critical Appraisal Checklist.

Publication bias was evaluated visually by inspecting funnel plots and statistically using Egger's regression test, with *p* < 0.05 considered indicative of significant publication bias. When publication bias was detected, we applied the trim‐and‐fill method to estimate its potential effect on the pooled prevalence. The robustness of the pooled estimates was further examined through sensitivity analyses using the logit transformation and a leave‐one‐out approach, in which each study was sequentially excluded to assess its influence on the overall results.

## 3 Results

### 3.1 Search Results

A total of 16,992 articles were identified via a search of the four aforementioned electronic databases. After removing duplicates, the titles and abstracts of 6312 articles were screened. Of these, 2572 met the inclusion criteria and were retained for full‐text review. Of the 2572 studies, 2197 were excluded because they were non‐original studies, conference abstracts, reviews, articles without full text, or studies with inappropriate data. Finally, 349 studies investigating *E. faecium* and 339 studies investigating *E. faecalis* met the eligibility criteria and were included in the systematic review and meta‐analysis (Abamecha et al. 2015; Abat et al. 2016; Abbassi et al. 2009; Abdelkareem et al. 2017; Adam et al. 2011; Adesida et al. 2017; Adeyemi et al. 2021; Ahirwar n.d.; Ahmad et al. 2024; Ahmed et al. 2023, 2015, 2021; Akhi et al. 2009; Al Bshabshe et al. 2024; Alduhaidhawi et al. 2022; Ali et al. 2009, 2014; Al‐Mijalli et al. 2022; Al‐Mohri et al. 2008; Ameri et al. 2009; Amuasi et al. 2023; Arhin et al. 2009; Armin et al. 2017; Arshadi et al. 2017, 2019; Asadian et al. 2016; Aslantaş 2019; O. et al. 2002; Aun et al. 2021; Aziz and Alabdali 2025; Aznar et al. 2012; Bantar et al. 2000; Belicová et al. 2007; Bell 2003; Ben Said et al. 2016; Bertrand et al. 2000; Bhat et al. 1997; Bhatt et al. 2015; Bhonsle et al. 2023; Biedenbach et al. 2007; Biggel et al. 2021; Billington et al. 2014; Billström et al. 2008; Birri et al. 2013; Biswas et al. 2013, 2016; Bitsori et al. 2005; Boccella et al. 2021; Bogut et al. 2021; Boisivon et al. 1997; Boost et al. 2004; Borcan et al. 2024; Bose et al. 2015; Bouchillon et al. 2004; Bourafa et al. 2016; Bouymajane et al. 2018; Bozo et al. 2025; Braga et al. 2013; Brandon and Dowzicky 2013; Brkic et al. 2017; Brown et al. 2008; Bulut et al. 2024; Busani et al. 2004; Butcu et al. 2011; Calderón‐Jaimes et al. 2003; Carrasco Calzada et al. 2023; Çay et al. 2023; Chan et al. 2008; Choi et al. 2022; Chotinantakul et al. 2018; Christenson et al. 1998; Cinquepalmi et al. 2013; Çitak et al. 2004; Claesson et al. 2007, 2009; Conceição et al. 2011; Coombs et al. 2018a, 2018b, 2016, 2020a, 2020b, 2024, 2023, 2013, 2014a, 2014b; Saba Çopur et al. 2016; Dadfarma et al. 2013; Dan et al. 1999; Daniel et al. 2017; Das et al. 2022a, 2022b, 2020, 2021; de Campos et al. 2015; Dehghani et al. 2022; Denisuik et al. 2019; Djahmi et al. 2012; Dolka et al. 2020; Donado‐Godoy et al. 2015; Dos Santos et al. 2020; Dowzicky and Chmelařová 2019; Dubey and Padhy 2015; Dungan and Bjorneberg 2021; Egan et al. 2020; Eichel et al. 2022; Ejaz 2019; Elmalı and Can 2018; El‐Masry et al. 2015; El‐Zamkan and Mohamed 2021; Emamie et al. 2023; Esmail et al. 2019; Falcone et al. 2019; Fernández‐Guerrero et al. 2002; Figueiredo et al. 2009; Fluit et al. 2000; Fontana et al. 1998; Fortun 2002; Freitas et al. 2018, 2006; Fujii et al. 2024; Furlaneto‐Maia et al. 2014; Garcia‐Llorens et al. 2024; Gawryszewska et al. 2016; Ge et al. 2022; Gebremariam et al. 2022; Ghoshal et al. 2006; Gomes et al. 2008; Gordon et al. 1992; Gray et al. 1994; Grudlewska‐Buda et al. 2023; Grüneberg and Hryniewicz 1998; Guerrero‐Ramos et al. 2016; Gümüş 2023; Hackney et al. 2019; Haldar et al. 2017; Hannaoui et al. 2016; Hansen et al. 2024; Harthug et al. 2000; Hazırolan 2021; Heidari et al. 2016; Hemapanpairoa et al. 2021; Henwood 2000; Hershberger et al. 2005; Hidano et al. 2015; Hirakata et al. 1997; Hitkova and Hristova 2019; Hornuss et al. 2024; Hota et al. 2025, 2024; Hsueh et al. 2001; Huang et al. 2024; Hufnagel et al. 2007; Huh et al. 2023; Huidrom et al. 2016; Iancu et al. 2023; Igbinosa and Beshiru 2019; Indikar et al. 2024; Iseppi et al. 2015; Jahansepas et al. 2018, 2020; Jain et al. 2011; Jannati et al. 2020, 2023; Japoni et al. 2009; Jia et al. 2014; Johnston and Jaykus 2004; Joudeh et al. 2023; Kaçmaz and Aksoy 2005; Mobarez et al. 2013; Kajihara et al. 2015; Kapoor et al. 2005; Karmarkar et al. 2004; Karna et al. 2019; Kasimoglu‐Dogru et al. 2010; Kateete et al. 2019, 2013; Kaur et al. 2015; Keepers et al. 2017; Kierzkowska et al. 2014; Kim et al. 2024, 2022, 2017, 2020, 2019, 2018; Kiruthiga et al. 2019; Kisaka et al. 2022; Kjerulf et al. 1996; Kobayashi 2000; Kobayashi et al. 2001; Kouidhi et al. 2011; Kraszewska et al. 2022; Kresken et al. 2009; Kuldeep Yadav 2023; Kuo et al. 2018; Kurtaran et al. 2011; Kuzdan et al. 2014; Kwon et al. 2013; Labecka et al. 2024; Lambiase et al. 2007; Landman et al. 1999; Lee et al. 2004, 2020, 2016, 2022; Li et al. 2007; Liao et al. 2024; Lim et al. 2006; Liu et al. 2023; Lo et al. 2011; Lopes et al. 2024; López‐Luis et al. 2021; Lu et al. 2024; Lübbert et al. 2015; Luty et al. 2020; Lytsy et al. 2017; Mackowiak 1989; Madani et al. 1999; Malik et al. 2022; Mališová et al. 2021; Malka et al. 2023; Manson et al. 2004; Marghmalek et al. 2021; Mastor et al. 2020; Mayoral‐Terán et al. 2020; Mengesha et al. 2024; Messele et al. 2022; Mishra et al. 2013; Moaddab and Rafi 2003; Mohamedali et al. 2017; Mohammadi et al. 2015; Moneoang and Bezuidenhout 2009; Monteiro and Santos 2020; Morris et al. 1995; Naidoo et al. 2023; Nasaj et al. 2016; Ngbede et al. 2023; Ngoi et al. 2021; Nicoletti et al. 2006; Nikmanesh et al. 2024; Nishimoto et al. 2005; Nocera et al. 2021; Noroozi et al. 2022; Noskin et al. 1995; Odoyo et al. 2023; Oliveira et al. 2016; Omar et al. 2004; Onaran et al. 2019; Oravcova et al. 2013; Ossiprandi et al. 2008; Ouyang et al. 2016; Palanisamy et al. 2013; Palazzo et al. 2011; Pan et al. 2024; Papadimitriou‐Olivgeris et al. 2016; Pérez‐Hernández et al. 2002; Perlada et al. 1997; Pesavento et al. 2014; Phukan et al. 2016; Piezzi et al. 2020; Pinholt et al. 2014; Poeta et al. 2005; Poulsen et al. 1999; Pourakbari et al. 2012; Pourshafie et al. 2008; Rao et al. 2020; Rezvani et al. 2016; Roy et al. 2025; Różańska et al. 2019; Sachin and Darji 2024; Sader et al. 2011; Saeidi et al. 2017; Saengsuwan et al. 2021; Saenhom et al. 2022; Said and Abdelmegeed 2019; Salah et al. 2008; Salamandane et al. 2023; Salem‐Bekhit et al. 2012; Sana et al. 2019; Sannathimmappa et al. 2023; Sattari‐Maraji et al. 2019; Schmitz et al. 1999; Seker et al. 2023; Sengupta et al. 2023; Seyfi et al. 2008; Gupta 2022; Shahi et al. 2020; Shahraki and Rabi 2017; Sharifi et al. 2012; Sheikh et al. 2019; Shete et al. 2019; Silva et al. 2017; Silvetti et al. 2019; Simonsen 2003; Somily et al. 2016; Baldaniya et al. 2024; Sreeja et al. 2012; Stolfa et al. 2021; Sun et al. 2009; Talebi et al. 2008; Tao et al. 2022; Tawfick et al. 2020; Thongkoom et al. 2012; Titze‐de‐Almeida et al. 2004; Toc et al. 2023; Todeschini et al. 2006; Todorić et al. 2023; Tollu and Ekin 2020; Trapti Sharma 2024; Tripathi et al. 2016; Udo et al. 2003; Ullah et al. 2015; von Baum et al. 1999; Walker et al. 2021; Wang et al. 2013, 2015; Wavare Sanjay and Sajjan 2015; Wei et al. 2014; Williams et al. 2024; Woźniak‐Biel et al. 2019; Yadav et al. 2017; Yadegarynia and Arab‐Mazar 2016; Ben Yahia et al. 2018; Yameen et al. 2013; Yang et al. 2024; Yangzom and Kumar Singh 2019; Yasliani et al. 2009; Zerrouki et al. 2021; Zhou et al. 2020a; Zolfaghar et al. 2025; Moemen et al. 2015; Mirzaii et al. 2023; Mhawesh and khudair 2022; Izayati Mastor et al. 2023; Littvik et al. 2006; Liese et al. 2019; Libisch et al. 2008; Leong et al. 2018; Ibrahim et al. 2011; Haghi et al. 2019; Huang et al. 2019; Hajiahmadi et al. 2016; Gu et al. 2009; Goel et al. 2016; Celik et al. 2014; Goudarzi et al. 2018; Howden et al. 2013; Karimaei et al. 2016; Bar et al. 2006; Banerjee and Anupurba 2015; Correia et al. 2014; Gorski et al. 2024; Cho et al. 2013; Di Pentima et al. 2014; Kuzma et al. 2022; Fatholahzadeh et al. 2006; Ducu et al. 2019; Alatorre‐Fernández et al. 2017; Cho et al. 2018; Alotaibi and Bukhari 2017; Iseppi et al. 2020; Doss Susai backiam et al. 2023; Lee et al. 2024; Mamishi et al. 2020; Christiansen et al. 2007; Jakovac et al. 2017; ALE and Mozafar 2005; Grayson et al. 1991; Düztaşlar and Gücükoğlu 2024; Dziri et al. 2016; Hafidh et al. 2024; Cheng et al. 2017; Ferdous et al. 2023; Bell et al. 1998; Dolna et al. 2008). Figure 1 presents the study selection process. Characteristics of included studies are presented in Supporting Information S1: Table S1/S2.

**Figure 1:**
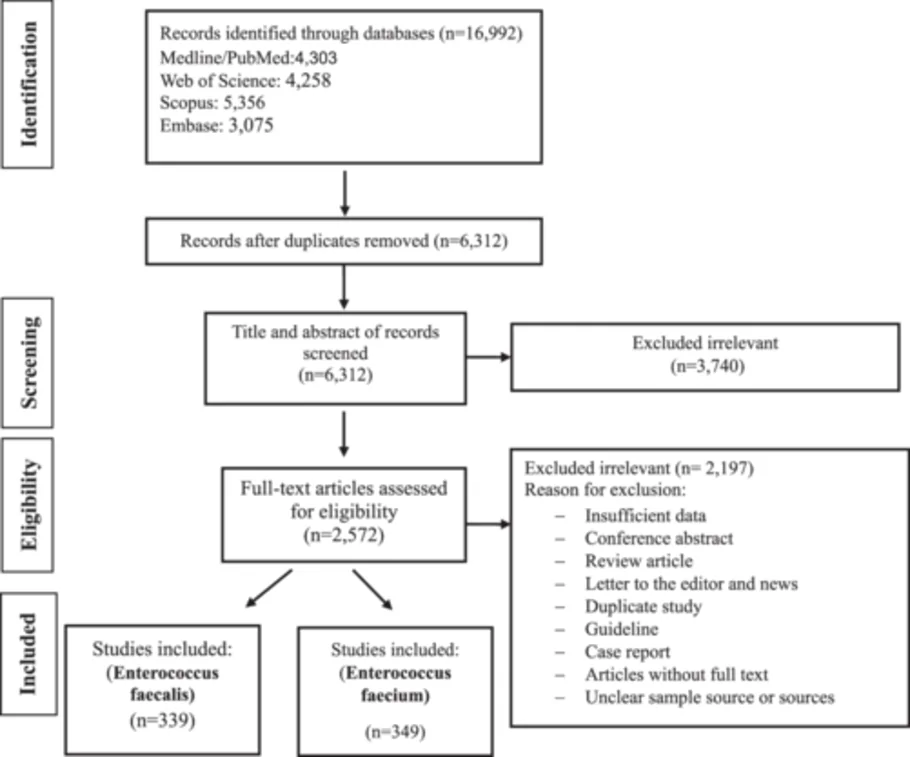
Flow chart of study selection for inclusion in the systematic review and meta‐analysis.

### 3.2 Prevalence of VREfs

We identified 2960 VREfs isolates among 144,481 *E. faecalis* isolates reported in 339 studies. Using a random‐effects model, the overall pooled prevalence of VREfs was estimated at 5% (95% CI: 3%–6%), with substantial heterogeneity observed among studies (*I* ^2^ = 95.6%, *p* < 0.001) (Supporting Information S1: Table S1). Also, the forest plot of the pooled prevalence of VREfs is presented in Supporting Information S1: File 2.

### 3.3 Subgroup Meta‐Analysis of VREfs Prevalence

Subgroup analyses were performed according to year of sample collection, continent, country, infection setting (hospital‐acquired vs. community‐acquired), hospital ward, isolate origin, source of isolates (clinical vs. non‐clinical), disease type, *E. faecalis* identification method, AST method used for VREfs detection, interpretive guideline, and study quality score.

Analysis by sample collection period showed a significant upward trend in the prevalence of VREfs. The pooled prevalence increased from 1% (95% CI: 0%–3%) before 2000 to 12% (95% CI: 3%–24%) during 2021–2025 (*p* = 0.0026) (Figure 2).

**Figure 2:**
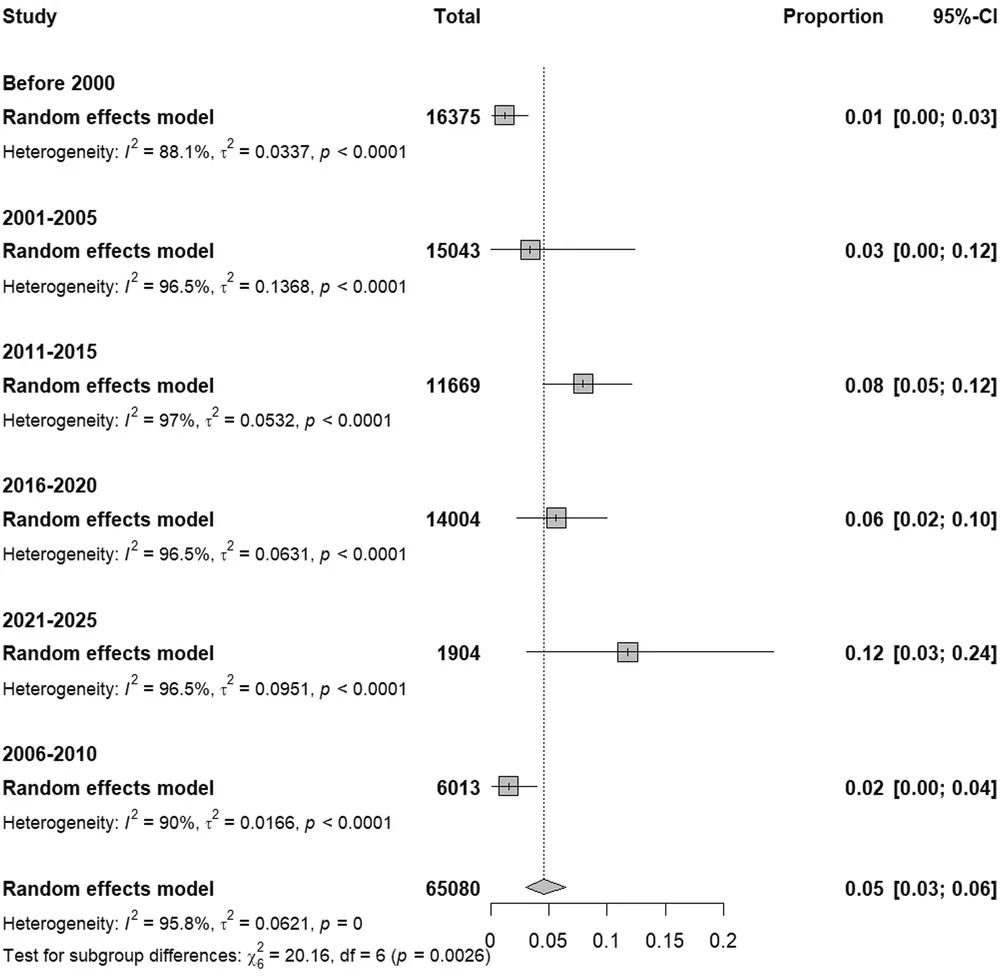
Subgroup meta‐analysis of vancomycin‐resistant *E. faecalis* prevalence by study period.

Subgroup analysis by continent revealed that Africa had the highest prevalence of VREfs isolates (13%; 95% CI: 4%–26%), whereas Oceania showed the lowest prevalence (0%; 95% CI: 0%–0%) (*p* < 0.001) (Figure 3).

**Figure 3:**
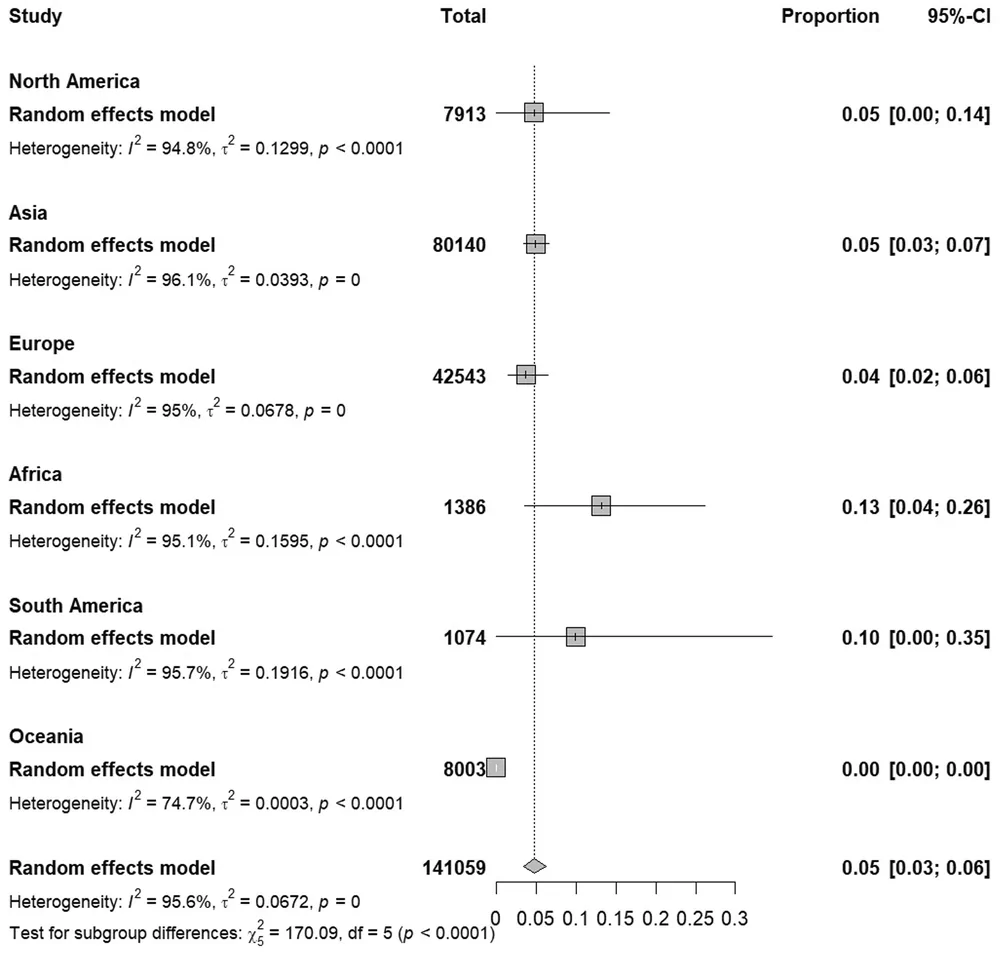
Subgroup meta‐analysis by continent of vancomycin‐resistant *E. faecalis* prevalence.

Country‐level analysis showed that Greece (50%; 95% CI: 0%–100%) and Slovakia (44%; 95% CI: 0%–100%) exhibited the highest pooled prevalence estimates of vancomycin‐resistant *E. faecalis* (*p* < 0.001) (Table 1).

**Table 1:** Subgroup analysis of vancomycin‐resistant *E. faecalis* prevalence by country.

Country	Studies (k)	Total *E. faecalis* (*n*)	VRE cases (e)	Pooled proportion (95% CI)
Algeria	3	209	15	0.0361 [0.0000–0.2251]
Australia	15	9994	39	0.0040 [0.0000–0.0140]
Bangladesh	3	233	24	0.0951 [0.0000–0.5087]
Brazil	6	548	46	0.1966 [0.0000–0.6170]
Canada	4	843	13	0.0084 [0.0000–0.0432]
China	15	36,083	199	0.0144 [0.0000–0.0542]
Croatia	4	345	18	0.0774 [0.0111– 0.1839]
Denmark	2	506	7	0.0100 [0.0019–0.0223]
Egypt	8	271	98	0.4052 [0.1198–0.7282]
Ethiopia	4	138	2	0.0062 [0.0000–0.0466]
Germany	6	2457	10	0.0000 [0.0000–0.0095]
Greece	2	25	12	0.4966 [0.0000–1.0000]
India	44	5850	716	0.0778 [0.0470–0.1145]
Iran	36	3995	293	0.0608 [0.0346–0.0927]
Iraq	5	212	47	0.2415 [0.1398–0.3592]
Italy	16	12,650	184	0.0129 [0.0000– 0.0605]
Japan	7	925	27	0.0089 [0.0000–0.0465]
Malaysia	6	354	30	0.0761 [0.0458–0.1124]
Morocco	2	56	8	0.0796 [0.0000–0.3182]
New Zealand	2	203	18	0.0410 [0.0000–0.2243]
Nigeria	4	111	35	0.3720 [0.0000–0.9197]
Pakistan	7	378	65	0.1674 [0.0457–0.3361]
Poland	11	3192	49	0.0216 [0.0003–0.0620]
Portugal	6	223	21	0.1887 [0.0000–0.6164]
Romania	5	1009	41	0.0709 [0.0151–0.1584]
Saudi Arabia	4	897	20	0.0000 [0.0000–0.0010]
Slovakia	2	58	9	0.4438 [0.0000–1.0000]
South Korea	12	11,975	114	0.0001 [0.0000–0.0007]
Spain	6	1172	4	0.0001 [0.0000–0.0089]
Sweden	2	692	14	0.0152 [0.0000–0.1085]
Taiwan	6	10,698	79	0.0066 [0.0000–0.0213]
Thailand	3	4923	7	0.0379 [0.0000–0.3196]
Tunisia	4	82	3	0.0103 [0.0000–0.0631]
Turkiye	18	1779	180	0.1025 [0.0157–0.2390]
Uganda	5	114	4	0.0741 [0.0000–0.4955]
United Kingdom	2	921	5	0.0052 [0.0000–0.0314]
USA	16	7058	248	0.0641 [0.0000–0.1989]

Regarding infection setting, the prevalence was 5% (95% CI: 2%–9%) among hospital‐acquired infections and 3% (95% CI: 0%–8%) among community‐acquired infections, although the difference was not statistically significant (*p* = 0.3167) (Figure 4).

**Figure 4:**
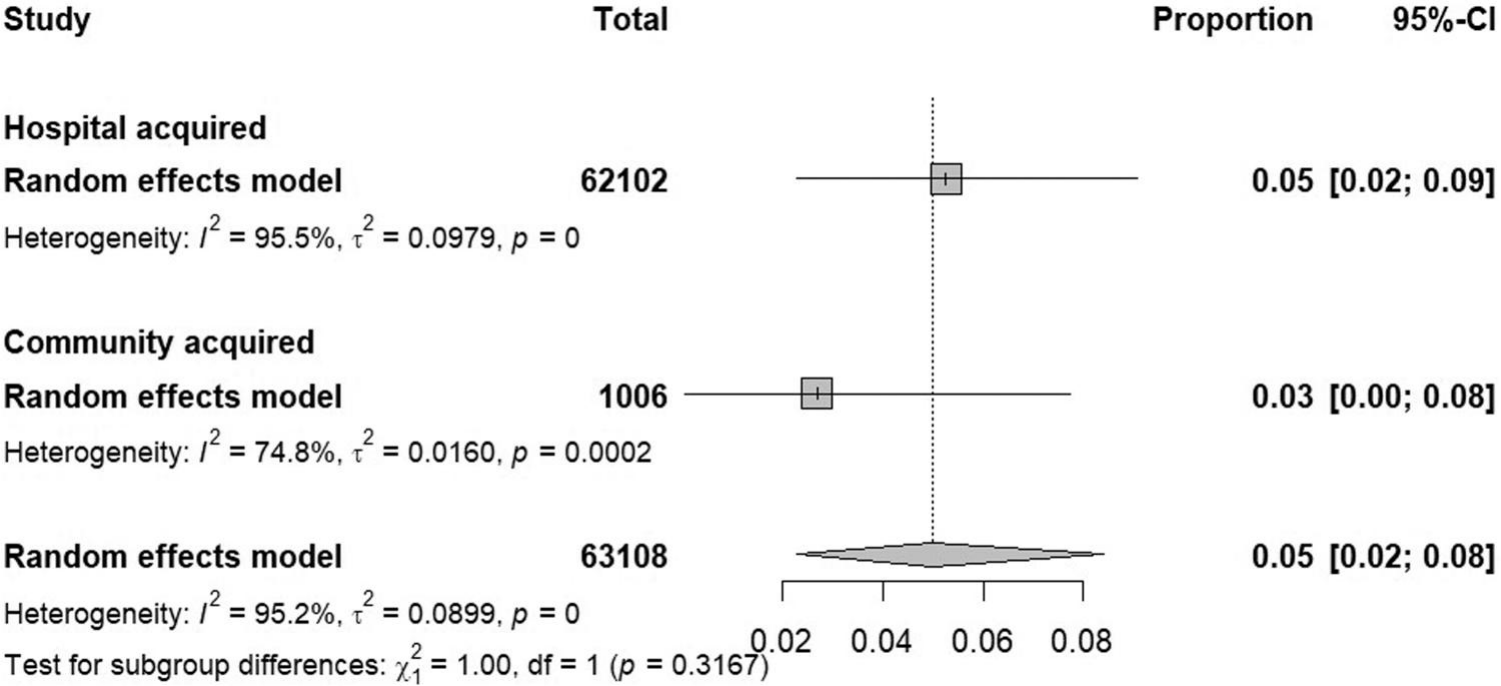
Subgroup meta‐analysis by infection setting of vancomycin‐resistant *E. faecalis* prevalence.

Analysis by hospital ward showed the highest prevalence in intensive care units (ICUs) (8%; 95% CI: 3%–33%), whereas hematology wards showed the lowest prevalence (0%; 95% CI: 0%–7%). However, no significant differences were observed between hospital wards (*p* = 0.8092) (Figure 5).

**Figure 5:**
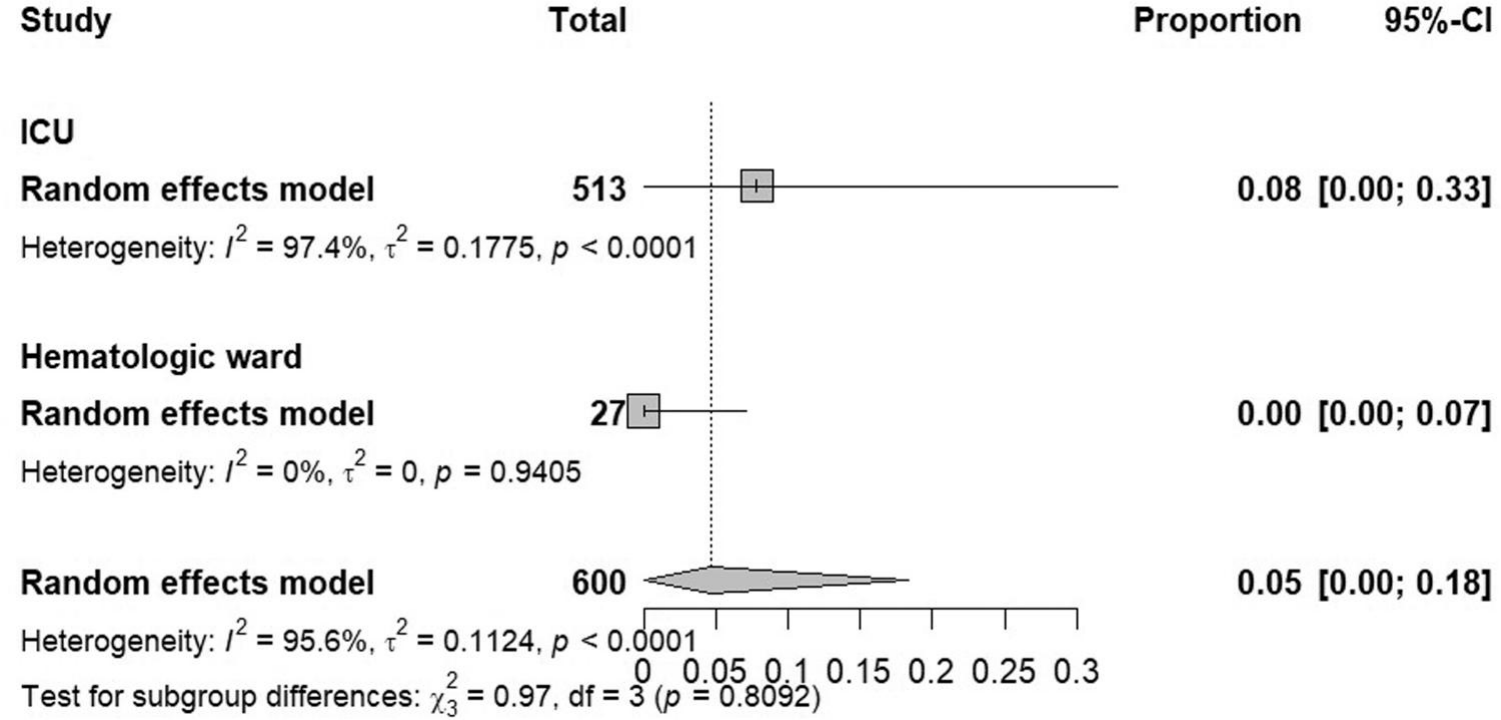
Subgroup meta‐analysis by hospital wards of vancomycin‐resistant *E. faecalis* prevalence.

Subgroup analysis based on isolate origin demonstrated that animal stool samples had the highest prevalence (40%; 95% CI: 0%–100%), whereas wound swab samples (0%; 95% CI: 0%–3%) and blood samples (0%; 95% CI: 0%–0%) showed the lowest prevalence estimates (*p* < 0.001) (Figure 6).

**Figure 6:**
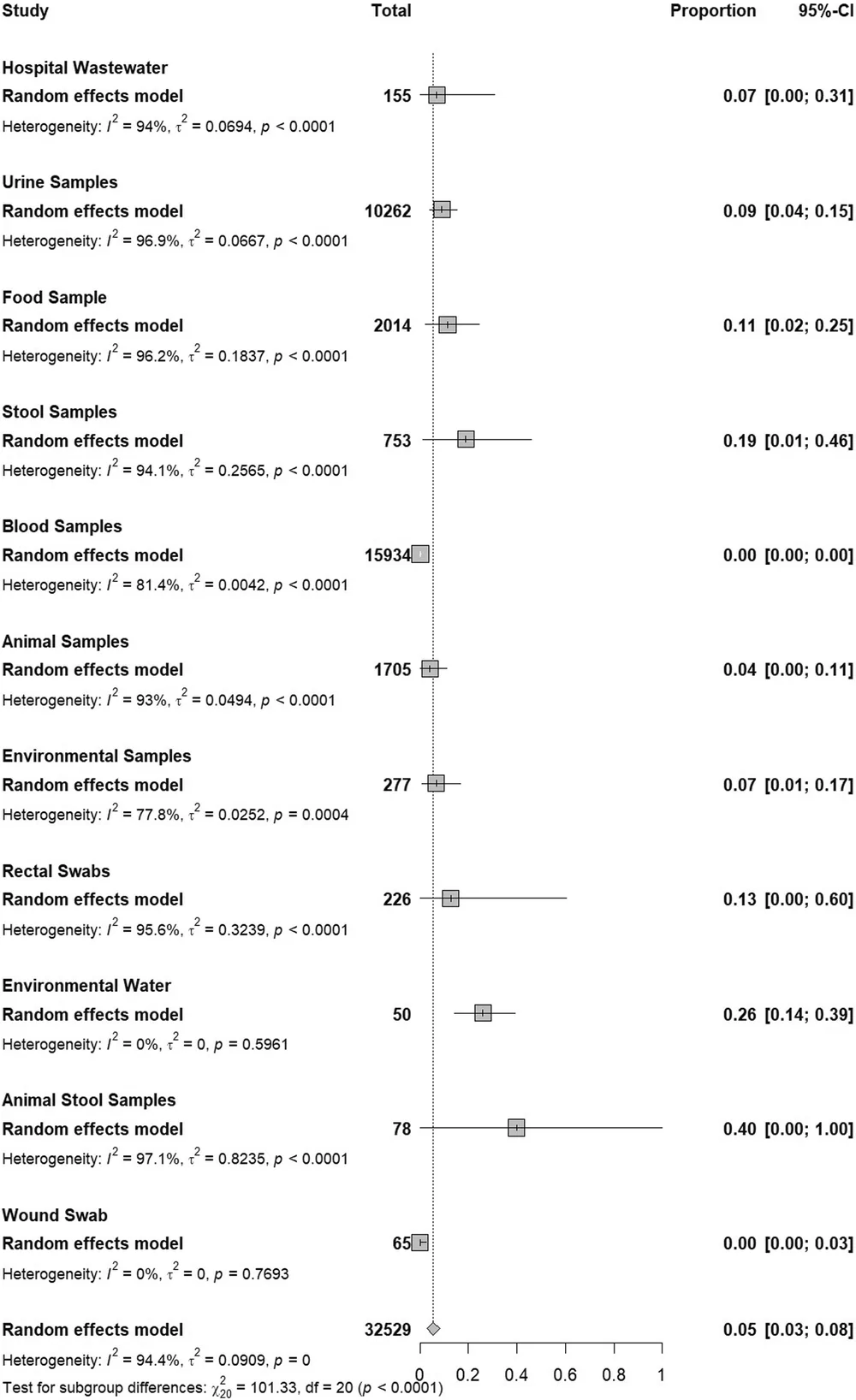
Subgroup meta‐analysis by isolate origin of vancomycin‐resistant *E. faecalis* prevalence.

When studies were categorized by isolate source, non‐clinical isolates had a higher prevalence (8%; 95% CI: 3%–13%) than clinical isolates (4%; 95% CI: 3%–6%) (*p* = 0.0267) (Figure 7).

**Figure 7:**
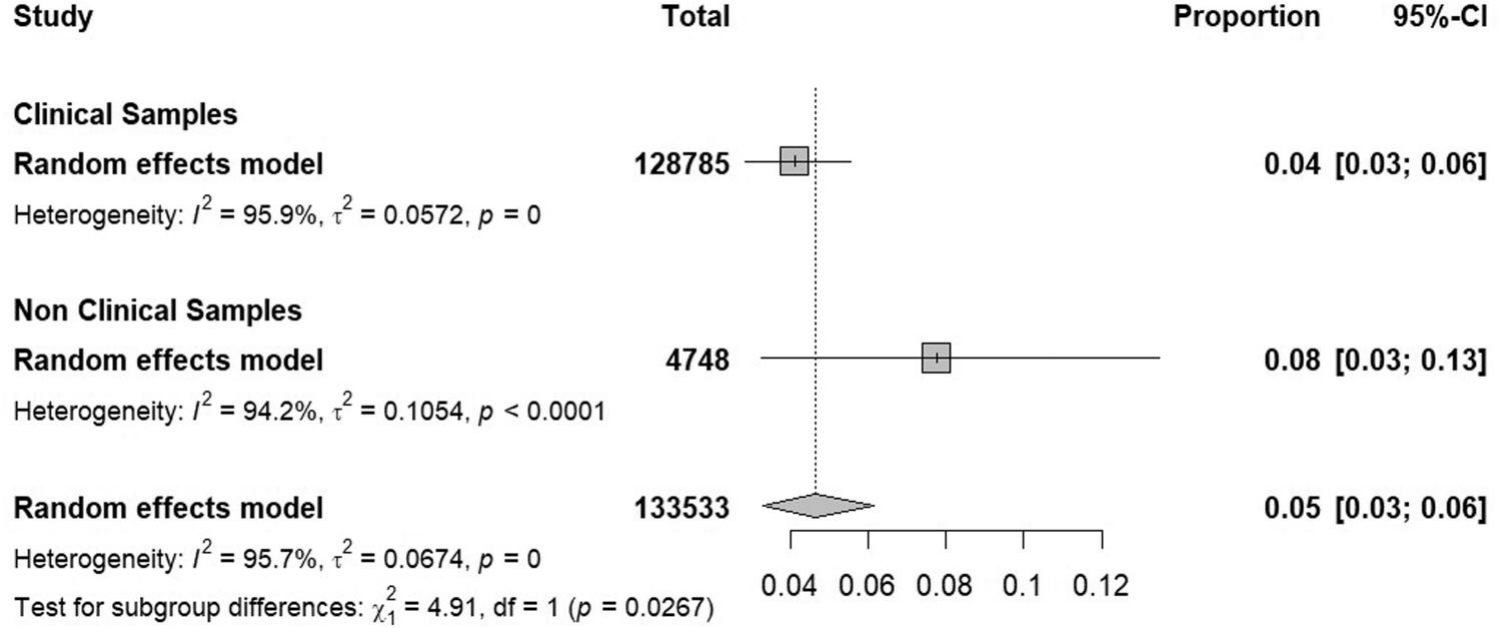
Subgroup meta‐analysis by source of isolates of vancomycin‐resistant *E. faecalis* prevalence.

Analysis by disease type revealed the highest prevalence among isolates associated with mastitis (65%; 95% CI: 22%–98%), followed by colonization (42%; 95% CI: 0%–95%) (*p* < 0.001) (Figure 8).

**Figure 8:**
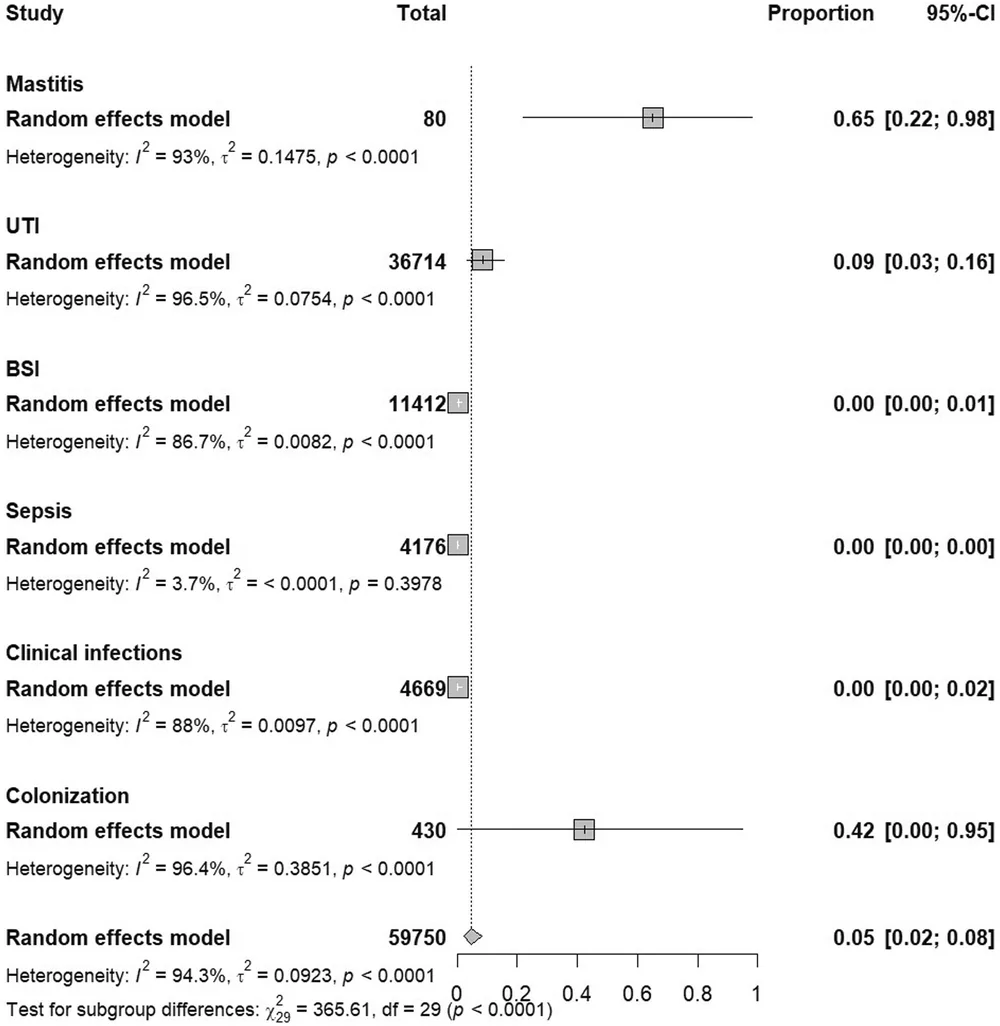
Subgroup meta‐analysis by disease type of vancomycin‐resistant *E. faecalis* prevalence.

Subgroup analysis according to the *E. faecalis* identification method showed that studies using biochemical tests combined with multiplex PCR reported the highest prevalence (10%; 95% CI: 1%–23%), whereas studies using PCR alone reported the lowest prevalence (1%; 95% CI: 0%–3%) (*p* < 0.001) (Figure 9).

**Figure 9:**
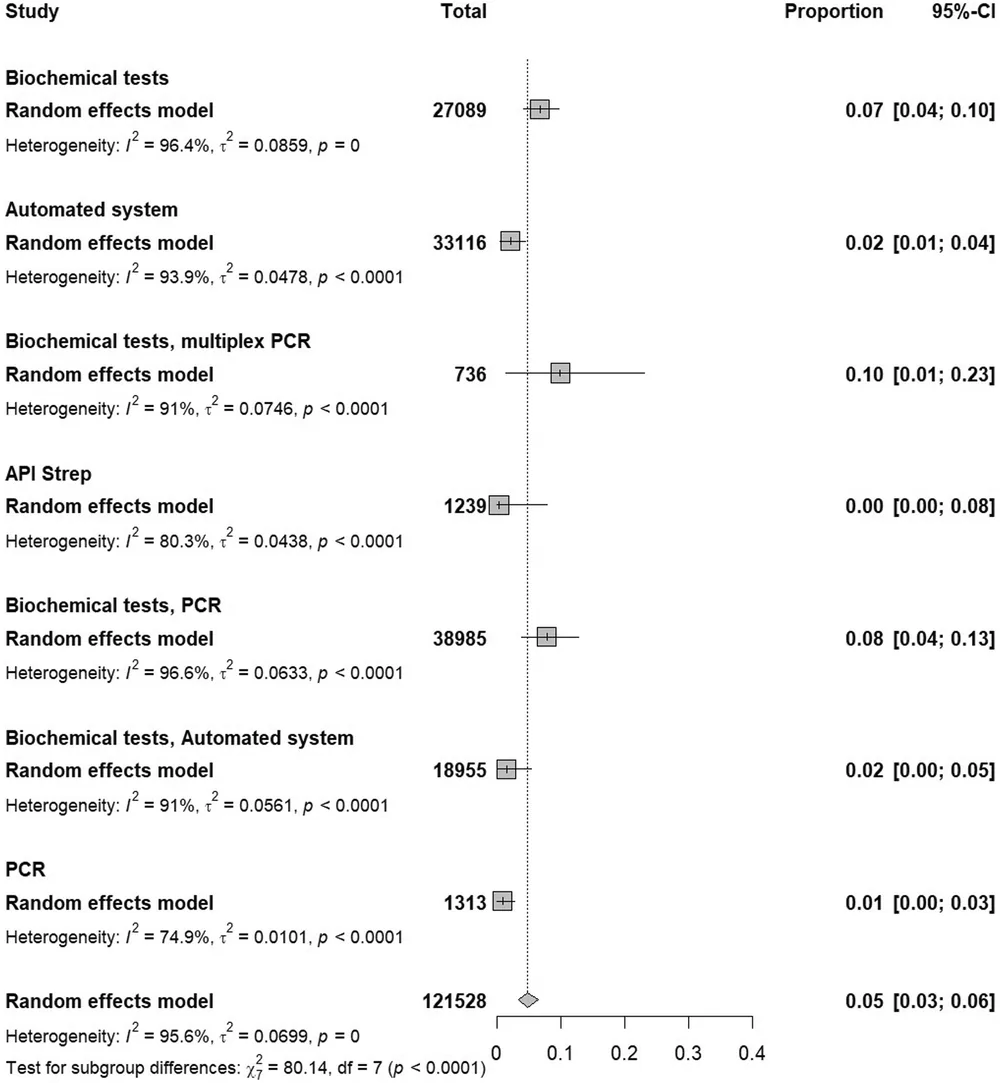
Subgroup meta‐analysis by *E. faecalis* diagnostic method.

Regarding AST methods, studies using disc diffusion combined with E‐test methods reported the highest prevalence (21%; 95% CI: 2%–51%), and studies employing broth microdilution combined with E‐test methods reported the lowest prevalence (0%; 95% CI: 0%–1%) (*p* < 0.001) (Figure 10).

**Figure 10:**
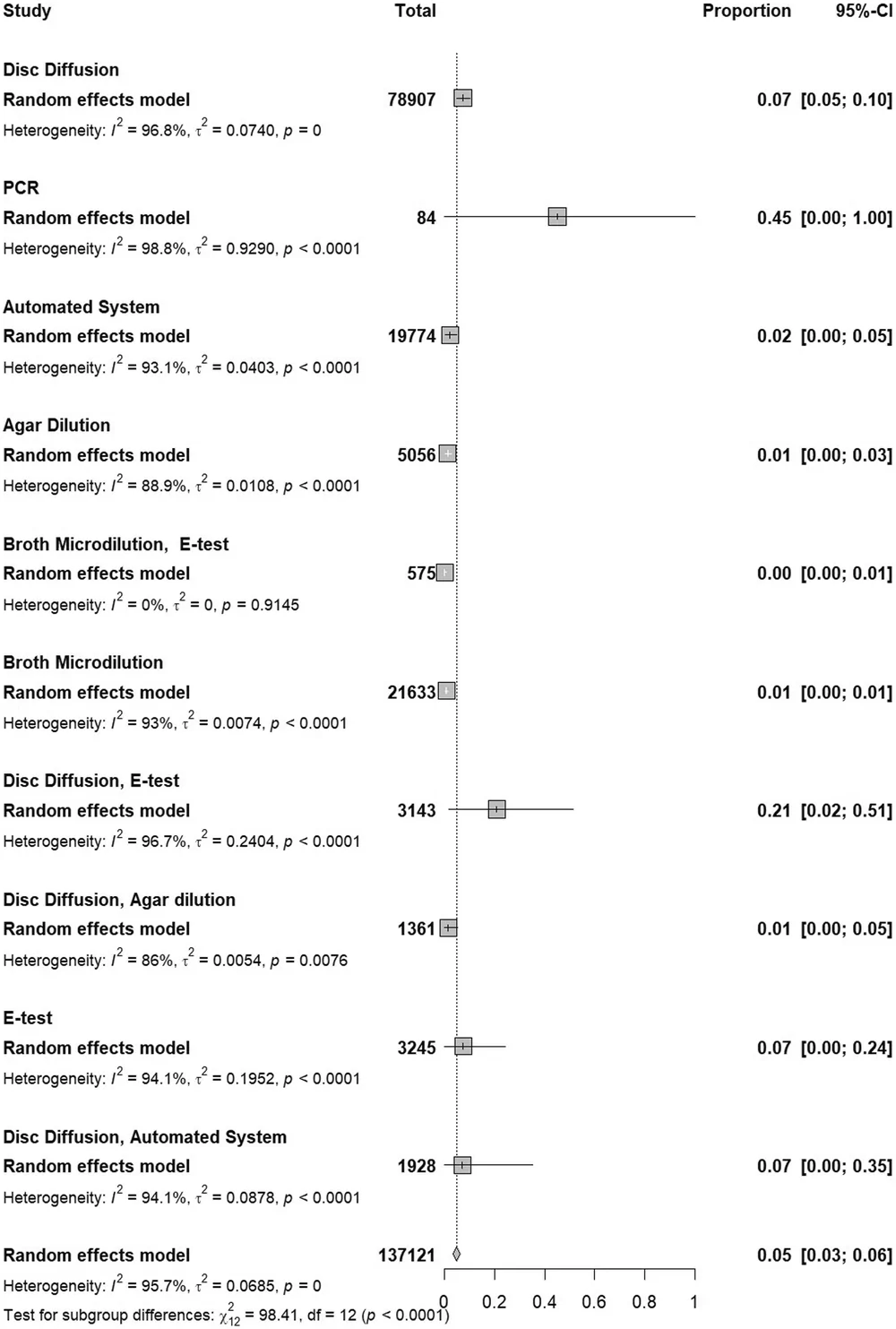
Subgroup meta‐analysis by antimicrobial susceptibility testing (AST) method used for vancomycin‐resistant *E. faecalis* detection.

Subgroup analysis based on interpretive guidelines indicated that studies applying CLSI criteria reported a pooled prevalence of 5% (95% CI: 4%–7%), representing the highest estimate among guideline categories (*p* < 0.001) (Figure 11).

**Figure 11:**
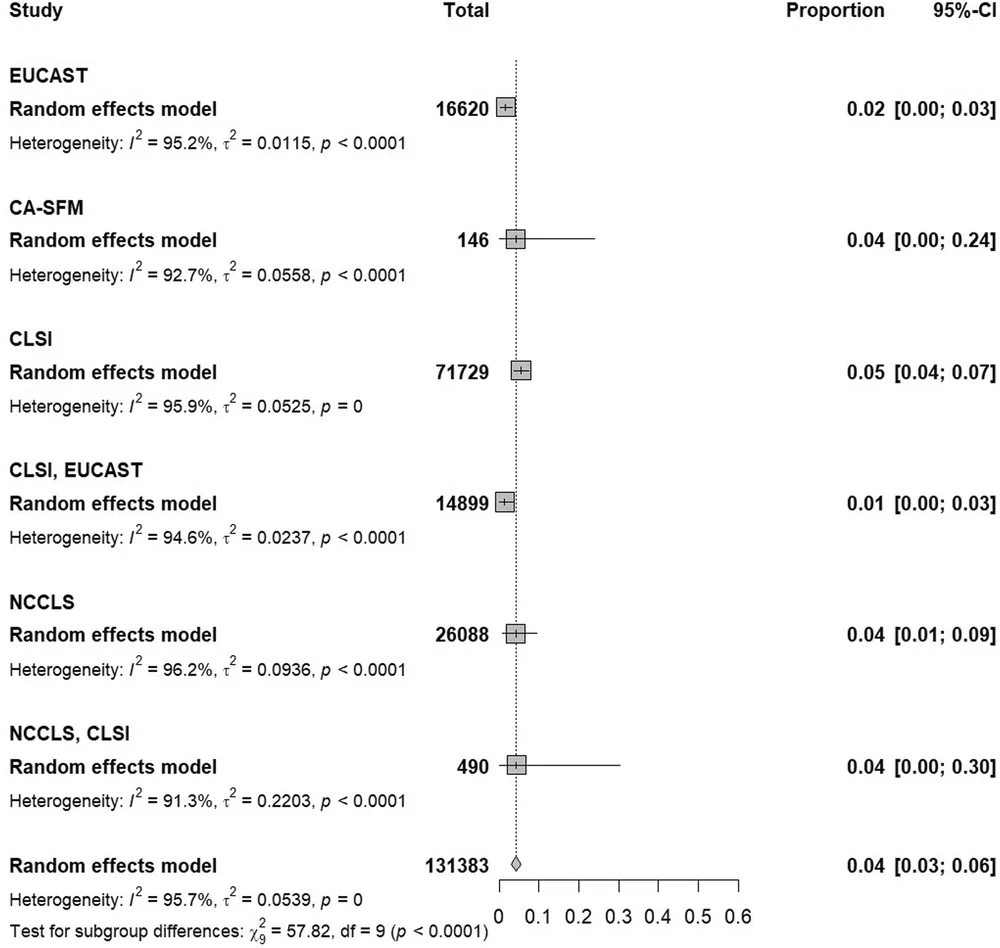
Subgroup meta‐analysis by guideline applied for vancomycin‐resistant *E. faecalis* prevalence.

According to the study quality assessment, moderate‐quality studies reported the highest prevalence (6%; 95% CI: 1%–14%). However, no statistically significant association was observed between study quality and prevalence estimates (*p* = 0.1977) (Figure 12).

**Figure 12:**
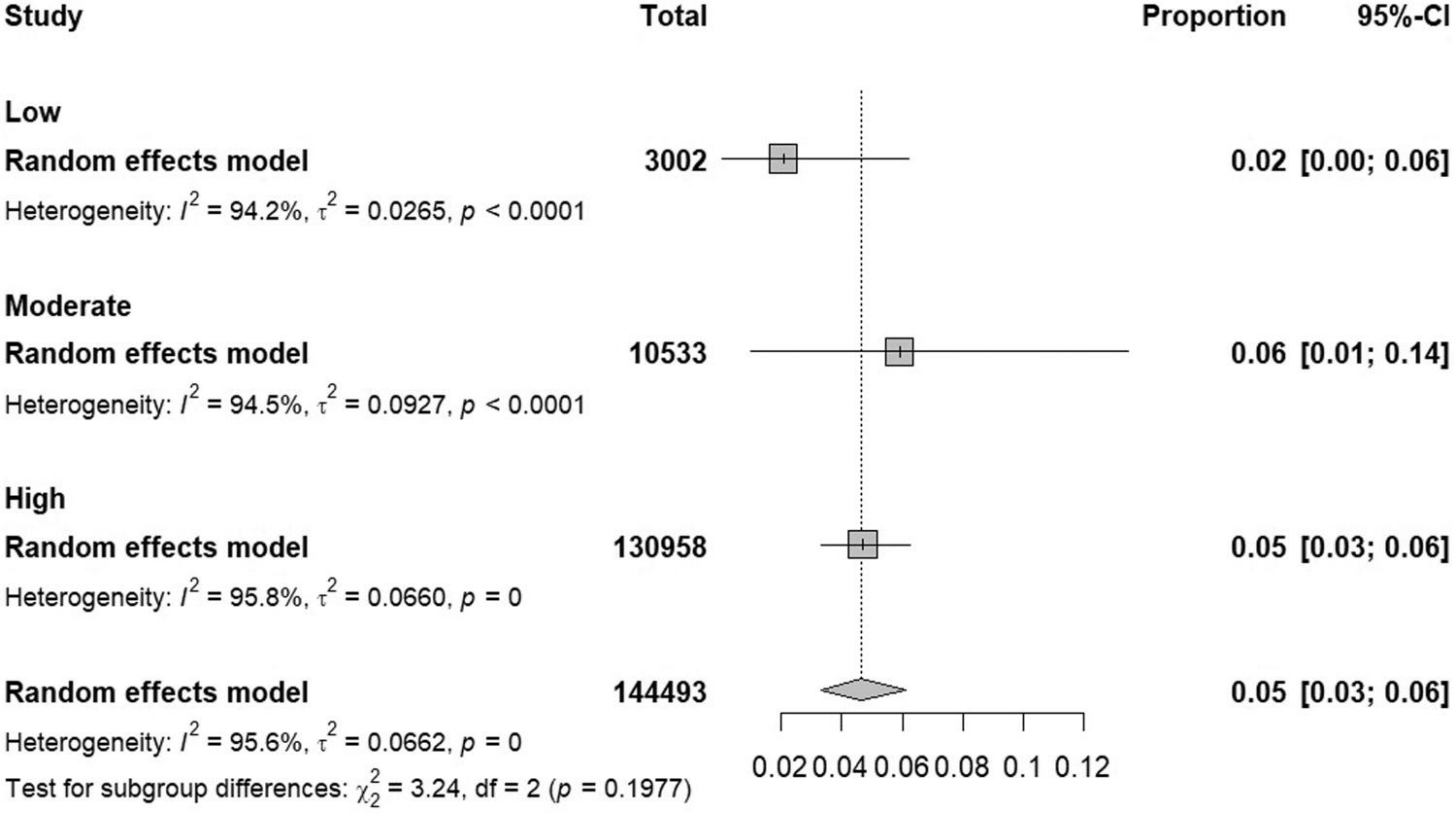
Subgroup meta‐analysis by quality score for vancomycin‐resistant *E. faecalis* prevalence.

### 3.4 Publication Bias and Sensitivity Analysis

We evaluated publication bias using a funnel plot (Figure 13). Egger's regression test indicated significant publication bias (*p* < 0.001). After adjustment using the trim‐and‐fill method, the pooled prevalence increased to 8% (95% CI: 6%–10%) (Figure 14).

**Figure 13:**
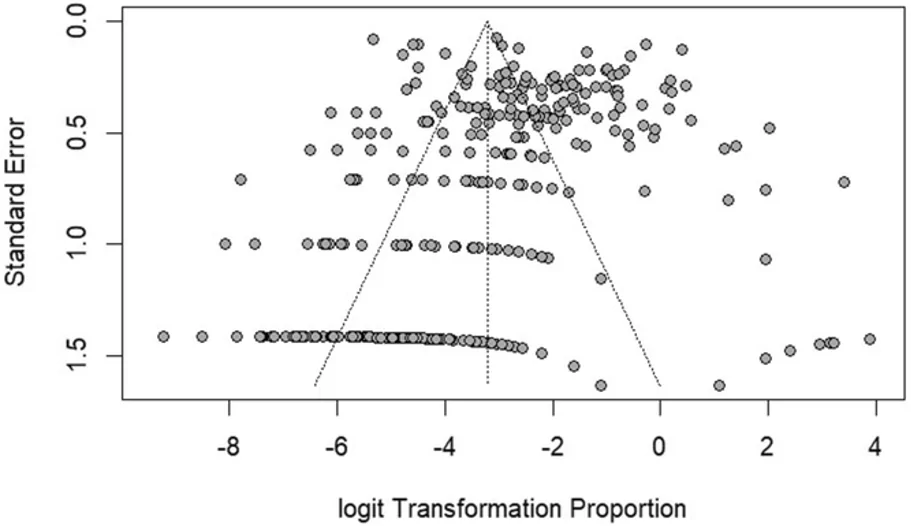
Funnel plot for assessment of publication bias in the included studies for vancomycin‐resistant *E. faecalis* prevalence.

**Figure 14:**
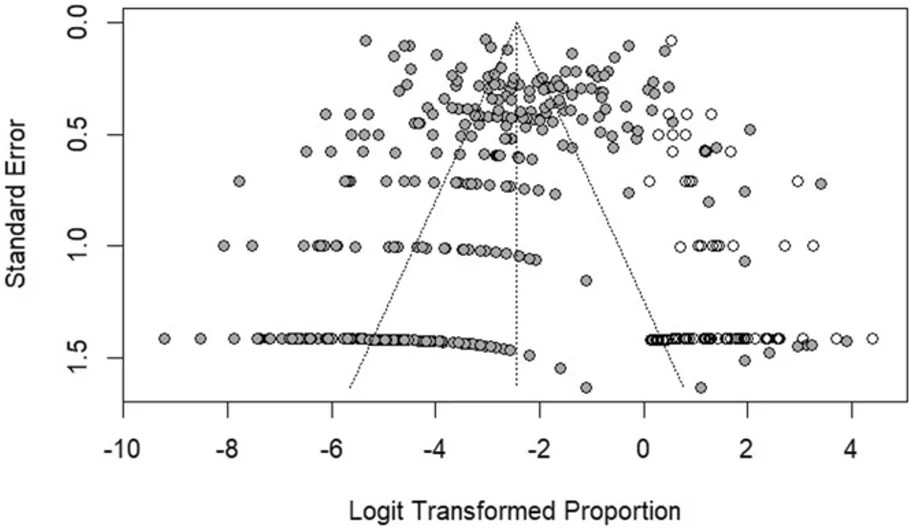
Trim‐and‐fill analysis to adjust for potential publication bias for vancomycin‐resistant *E. faecalis* prevalence.

Sensitivity analysis using the leave‐one‐out approach demonstrated that excluding any single study did not substantially affect the pooled prevalence estimate, confirming the robustness and stability of the overall findings.

### 3.5 Prevalence of VREfm

A total of 18,679 VREfm isolates were identified among 94,790 *E. faecium* isolates reported in 349 studies. Using a random‐effects model, the overall pooled prevalence of VREfm was estimated at 25% (95% CI: 21%–29%), with substantial heterogeneity observed among studies (*I* ^2^ = 99.3%, *p* < 0.001) (Supporting Information S1: Table S2). Also, the forest plot of the pooled prevalence of VREfm is presented in Supporting File 3).

### 3.6 Subgroup Meta‐Analysis of VREfm Prevalence

Analysis by sample collection period showed a significant trend in the prevalence of VREfm. The pooled prevalence ranged from 11% (95% CI: 5%–19%) before 2000 to 29% (95% CI: 18%–43%) during 2021–2025 (*p* = 0.0095) (Figure 15).

**Figure 15:**
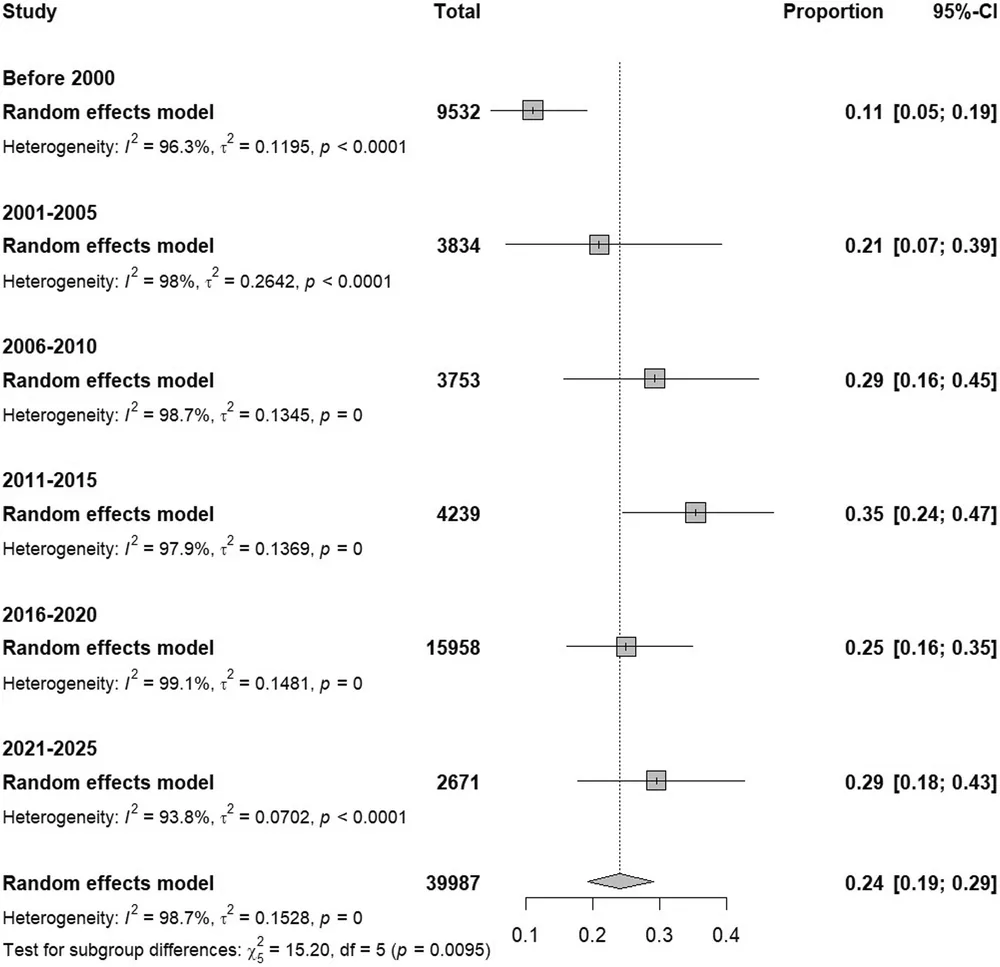
Subgroup meta‐analysis of vancomycin‐resistant *E. faecium* prevalence by study period.

Subgroup analysis by continent revealed that South America had the highest prevalence of VREfm isolates (57%; 95% CI: 26%–86%), whereas Africa showed the lowest prevalence (5%; 95% CI: 1%–12%) (*p* < 0.001) (Figure 16).

**Figure 16:**
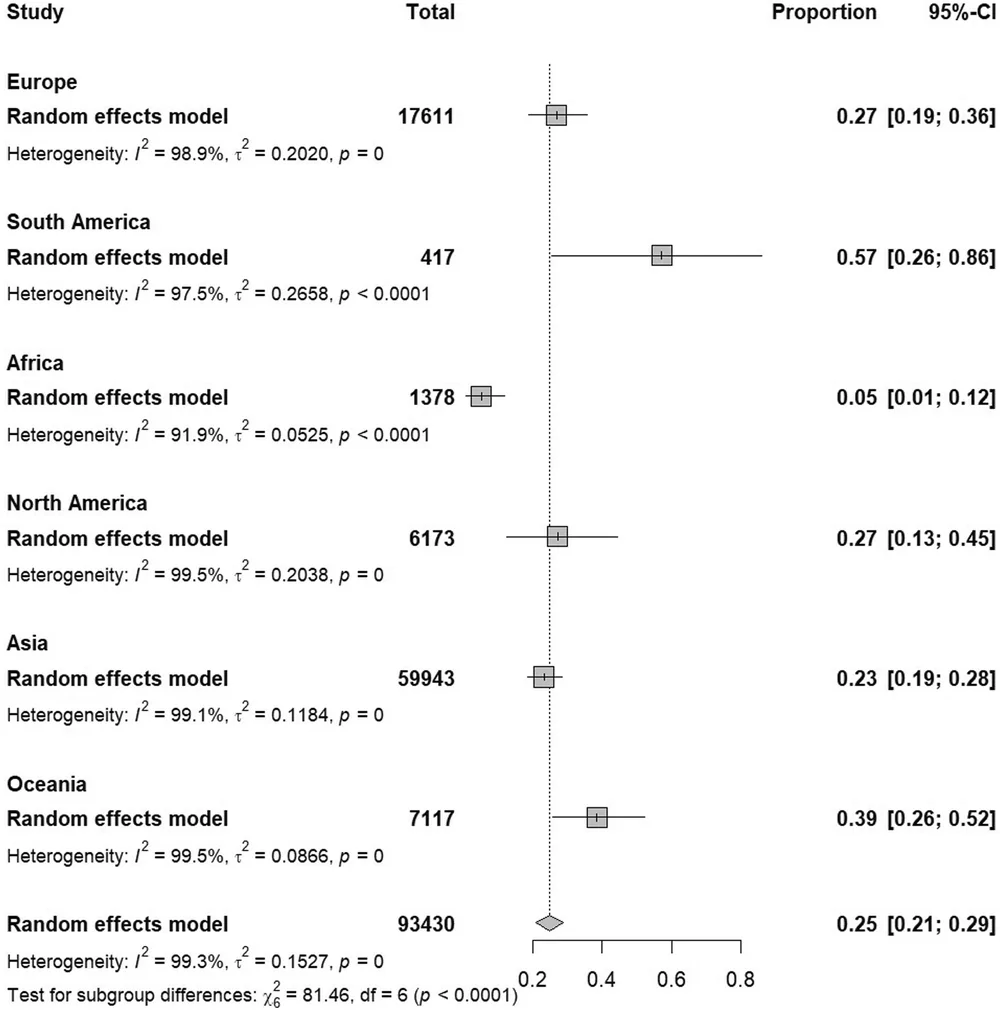
Subgroup meta‐analysis by continent of vancomycin‐resistant *E. faecium* prevalence.

Country‐level analysis showed that Ireland (100%; 95% CI: 95%–100%) and Argentina (74%; 95% CI: 2%–100%) exhibited the highest pooled prevalence estimates of VREfm (*p* < 0.001) (Table 2).

**Table 2:** Subgroup analysis of vancomycin‐resistant *Enterococcus faecium* prevalence by country.

Country	Studies (k)	Total *E. faecium* (*n*)	VRE cases (e)	Pooled proportion (95% CI)
Algeria	3	129	15	0.0408 [0.0000 – 0.2175]
Argentina	2	46	26	0.7375 [0.0155 – 1.0000]
Australia	19	7327	4041	0.4459 [0.2957 – 0.6014]
Bangladesh	2	15	1	0.0419 [0.0000 – 0.2609]
Brazil	8	345	154	0.6265 [0.2731 – 0.9209]
Canada	5	478	85	0.2696 [0.0020 – 0.7098]
China	15	31,457	609	0.1141 [0.0155 – 0.2735]
Croatia	3	228	99	0.5525 [0.2593 – 0.8279]
Czech Republic	2	838	82	0.0943 [0.0749 – 0.1156]
Denmark	4	2457	114	0.0396 [0.0075 – 0.0896]
Egypt	5	131	35	0.3143 [0.0937 – 0.5856]
Ethiopia	3	125	17	0.0825 [0.0033 – 0.2217]
France	3	578	17	0.2683 [0.0000 – 0.9847]
Germany	8	3541	1664	0.5432 [0.1642 – 0.8954]
India	36	2325	378	0.1421 [0.0973 – 0.1927]
Iran	41	3632	1242	0.3729 [0.2632 – 0.4892]
Iraq	4	171	35	0.2034 [0.1372 – 0.2780]
Ireland	2	39	39	1.0000 [0.9508 – 1.0000]
Italy	13	2563	278	0.0983 [0.0257 – 0.2027]
Japan	7	304	24	0.0389 [0.0000 – 0.1728]
Malaysia	4	102	7	0.1698 [0.0000 – 0.7080]
Mexico	5	347	173	0.3576 [0.1275 – 0.6278]
Morocco	2	58	13	0.1857 [0.0772 – 0.3179]
New Zealand	2	192	5	0.0549 [0.0000 – 0.3064]
Nigeria	3	240	67	0.1421 [0.0032 – 0.3971]
Norway	2	150	4	0.0000 [0.0000 – 0.0000]
Pakistan	6	204	40	0.1542 [0.0398 – 0.3130]
Poland	10	985	53	0.0858 [0.0351 – 0.1509]
Portugal	8	419	29	0.1676 [0.0000 – 0.5432]
Romania	5	259	103	0.4147 [0.2342 – 0.6071]
Saudi Arabia	5	442	194	0.5033 [0.1307 – 0.8741]
Slovakia	2	205	27	0.4679 [0.0000 – 1.0000]
South Africa	2	285	3	0.0077 [0.0000 – 0.0549]
South Korea	15	10,272	2148	0.2211 [0.1104 – 0.3520]
Spain	6	241	17	0.1478 [0.0000 – 0.5932]
Sweden	2	301	66	0.5494 [0.0000 – 1.0000]
Switzerland	2	2179	36	0.4431 [0.0000 – 1.0000]
Taiwan	9	5725	2774	0.3279 [0.1061 – 0.5981]
Thailand	5	4427	376	0.4135 [0.0168 – 0.9025]
Tunisia	4	292	0	0.0000 [0.0000 – 0.0000]
Turkey	19	1197	316	0.3030 [0.1533 – 0.4756]
Uganda	4	46	0	0.0000 [0.0000 – 0.0189]
United Kingdom	3	647	147	0.1221 [0.0011 – 0.3730]
USA	16	5345	2160	0.2504 [0.0647 – 0.4943]

Regarding infection setting, the prevalence was 29% (95% CI: 21%–38%) among hospital‐acquired infections and 1% (95% CI: 0%–7%) among community‐acquired infections (*p* < 0.001) (Figure 17).

**Figure 17:**
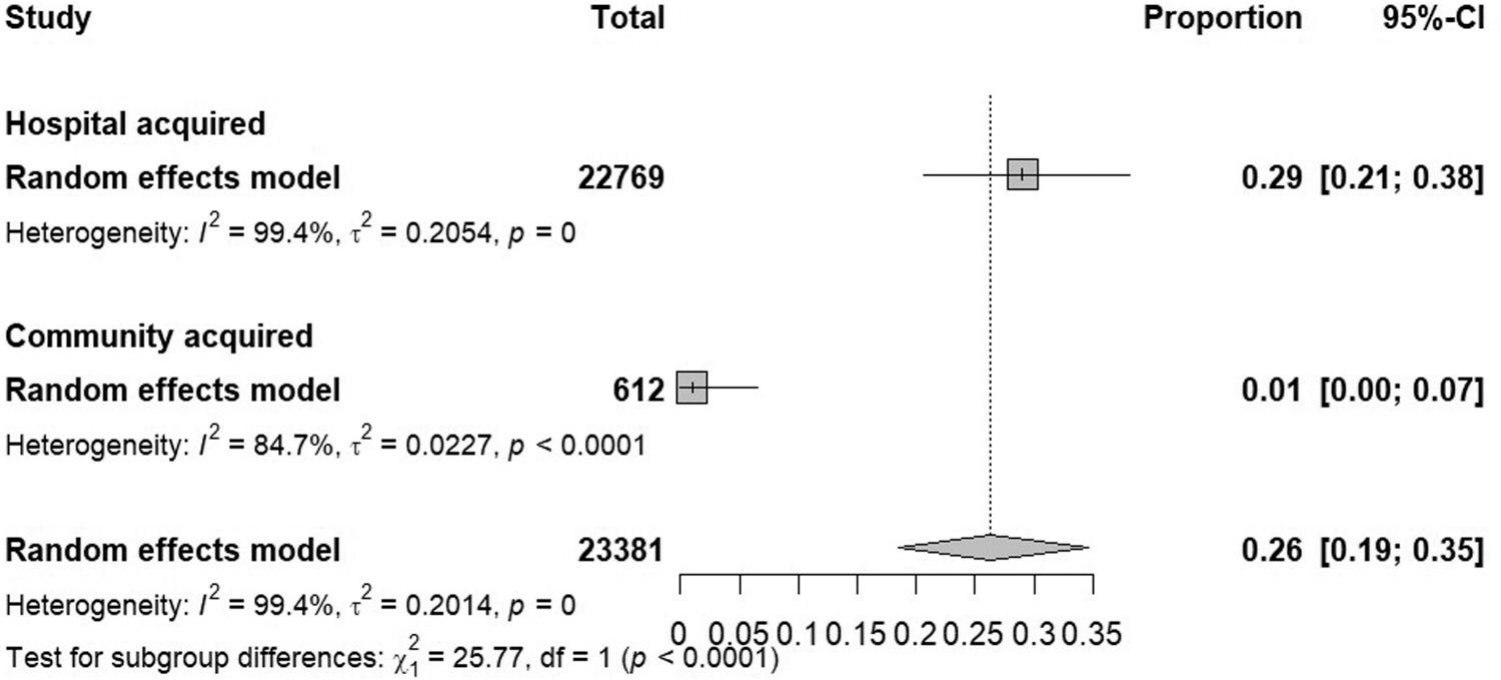
Subgroup meta‐analysis by infection setting of vancomycin‐resistant *E. faecium* prevalence.

Analysis by hospital ward showed the highest prevalence in the hematological ward (49%; 95% CI: 31%–66%), whereas the ICU (31%; 95% CI: 9%–58%) showed the lowest prevalence (*p* < 0.001) (Figure 18).

**Figure 18:**
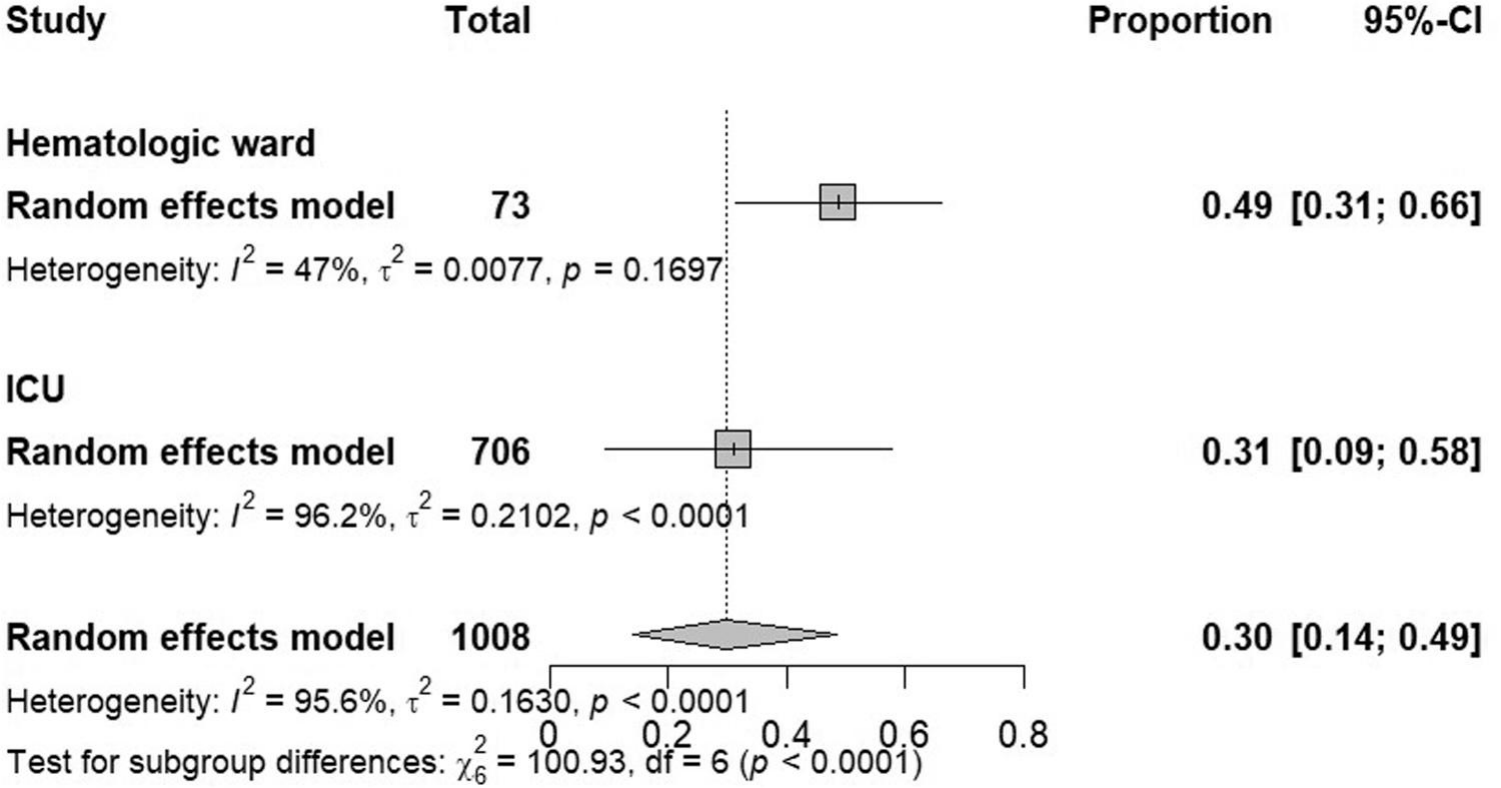
Subgroup meta‐analysis by hospital wards of vancomycin‐resistant *E. faecium* prevalence.

Subgroup analysis by isolate origin showed that rectal swabs (76%; 95% CI: 38%–99%) and hospital environment samples (74%; 95% CI: 33%–99%) had the highest prevalence (*p* < 0.001) (Figure 19).

**Figure 19:**
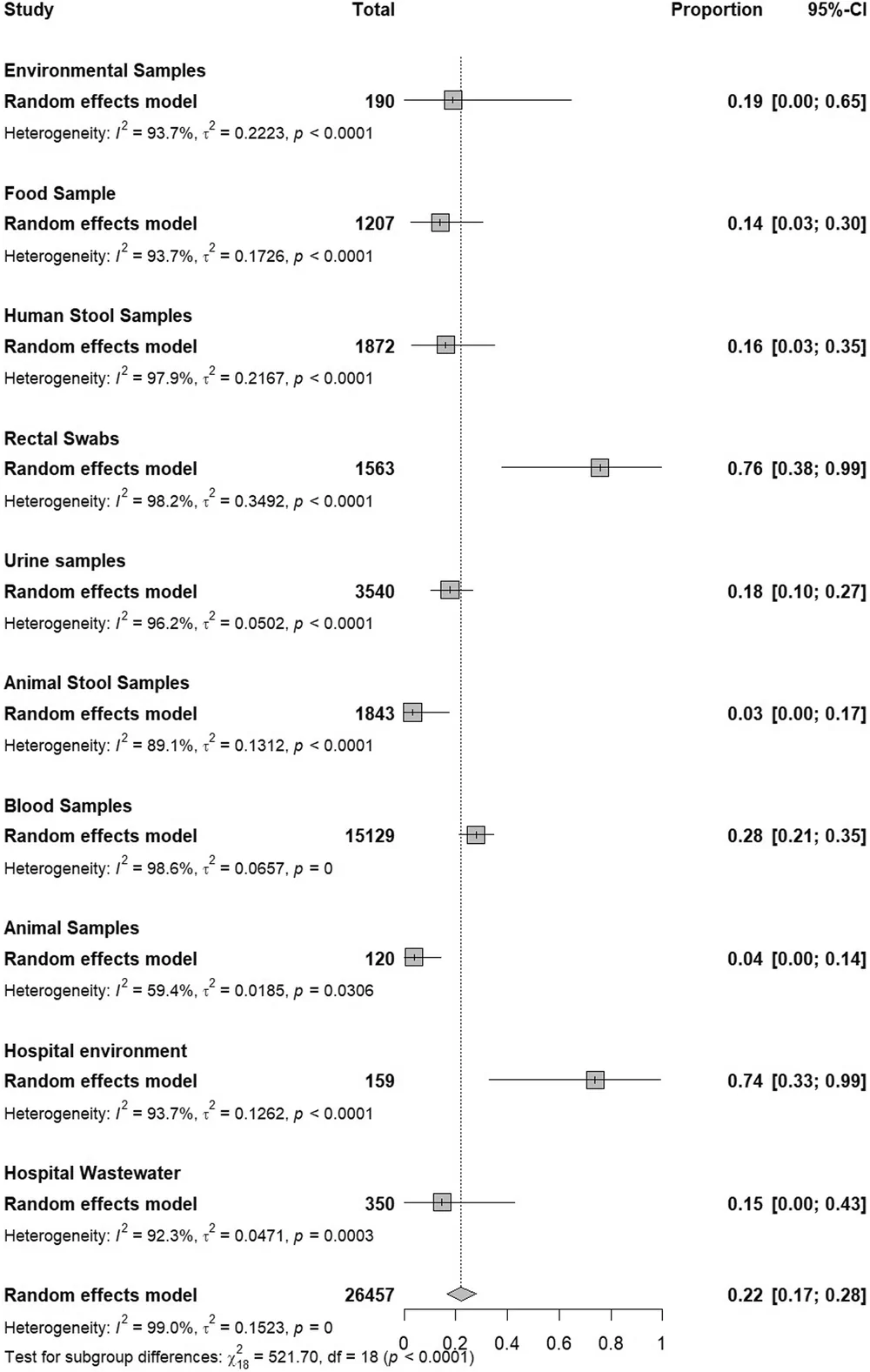
Subgroup meta‐analysis by isolate origin of vancomycin‐resistant *E. faecium* prevalence.

Analysis by disease type revealed the highest prevalence among isolates associated with nosocomial infection (61%; 95% CI: 17%–96%), and urinary tract infection (UTI) had the lowest (24%; 95% CI: 10%–40%) (*p* < 0.001) (Figure 20).

**Figure 20:**
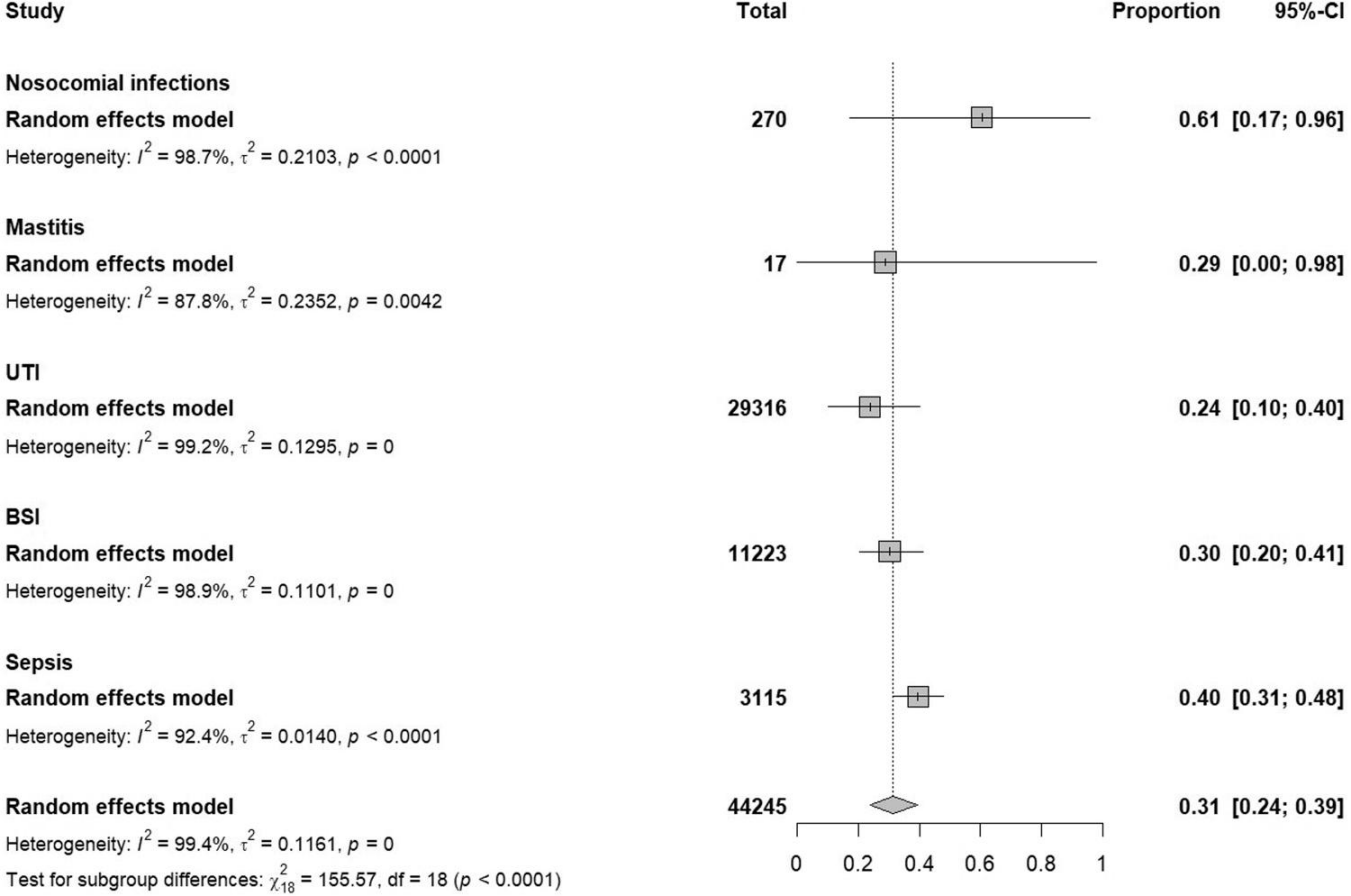
Subgroup meta‐analysis by disease type of vancomycin‐resistant *E. faecium* prevalence.

Subgroup analysis according to the *E. faecium* identification method showed that studies using biochemical tests combined with the PCR test reported the highest prevalence (42%; 95% CI: 27%–58%), whereas studies using API Strep reported the lowest prevalence (3%; 95% CI: 0%–14%) (*p* < 0.001) (Figure 21).

**Figure 21:**
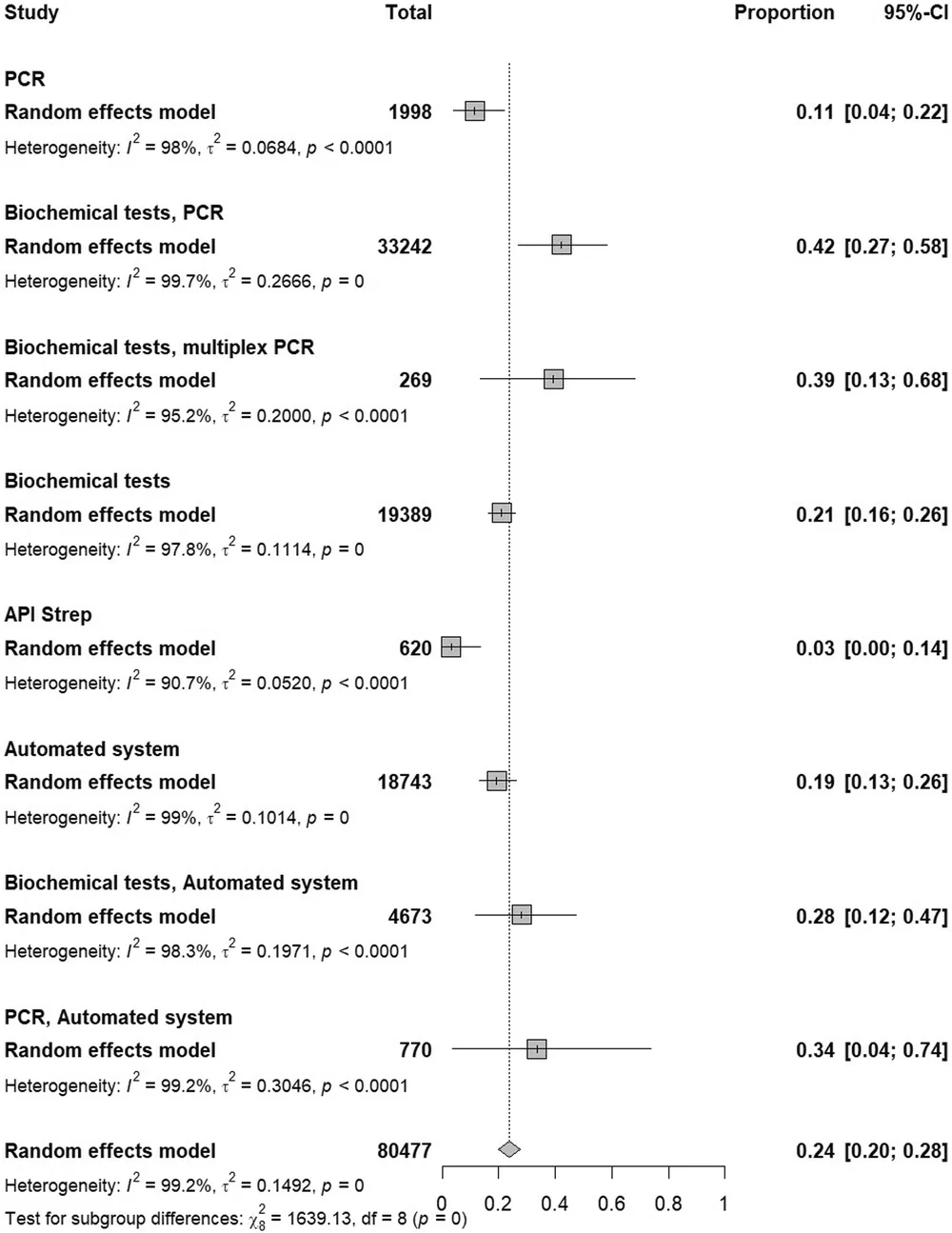
Subgroup meta‐analysis by *E. faecium* diagnostic method.

Regarding AST methods, studies using the E‑test reported the highest prevalence (64%; 95% CI: 39%–85%), followed by studies employing disc diffusion combined with E‐test methods (35%; 95% CI: 12%–62%) (*p* < 0.001) (Figure 22).

**Figure 22:**
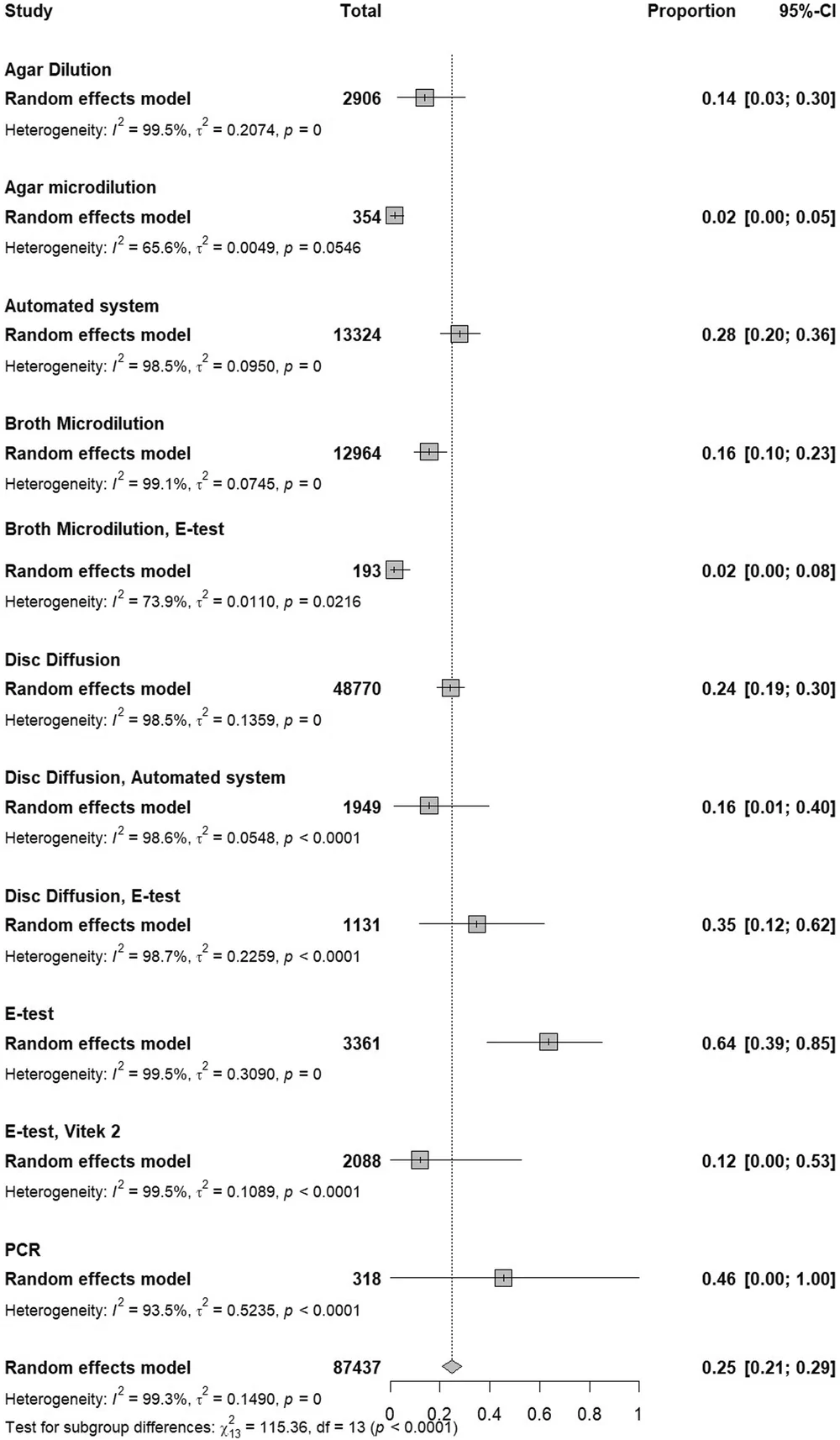
Subgroup meta‐analysis by antimicrobial susceptibility testing (AST) method used for vancomycin‐resistant *E. faecium* detection.

Subgroup analysis based on interpretive guidelines indicated that studies applying EUCAST criteria reported a pooled prevalence of 36% (95% CI: 22%–52%), representing the highest estimate among guideline categories (*p* < 0.001) (Figure 23).

**Figure 23:**
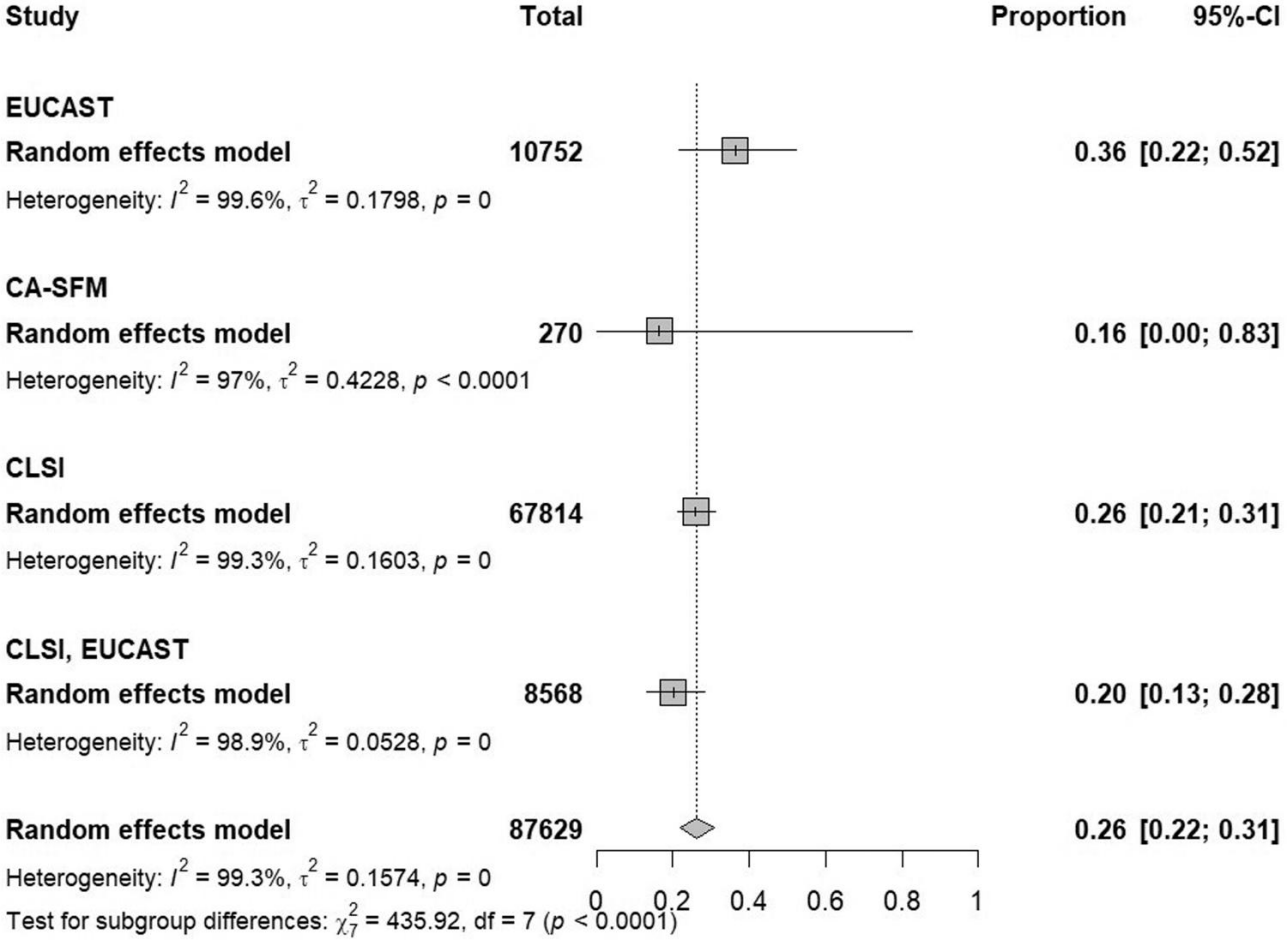
Subgroup meta‐analysis by guideline applied for vancomycin‐resistant *E. faecium* prevalence.

According to the study quality assessment, moderate‐quality studies reported the highest prevalence (29%; 95% CI: 16%–43%), while low‐quality studies had the lowest (6%; 95% CI: 1%–15%) (*p* = 0.0008) (Figure 24).

**Figure 24:**
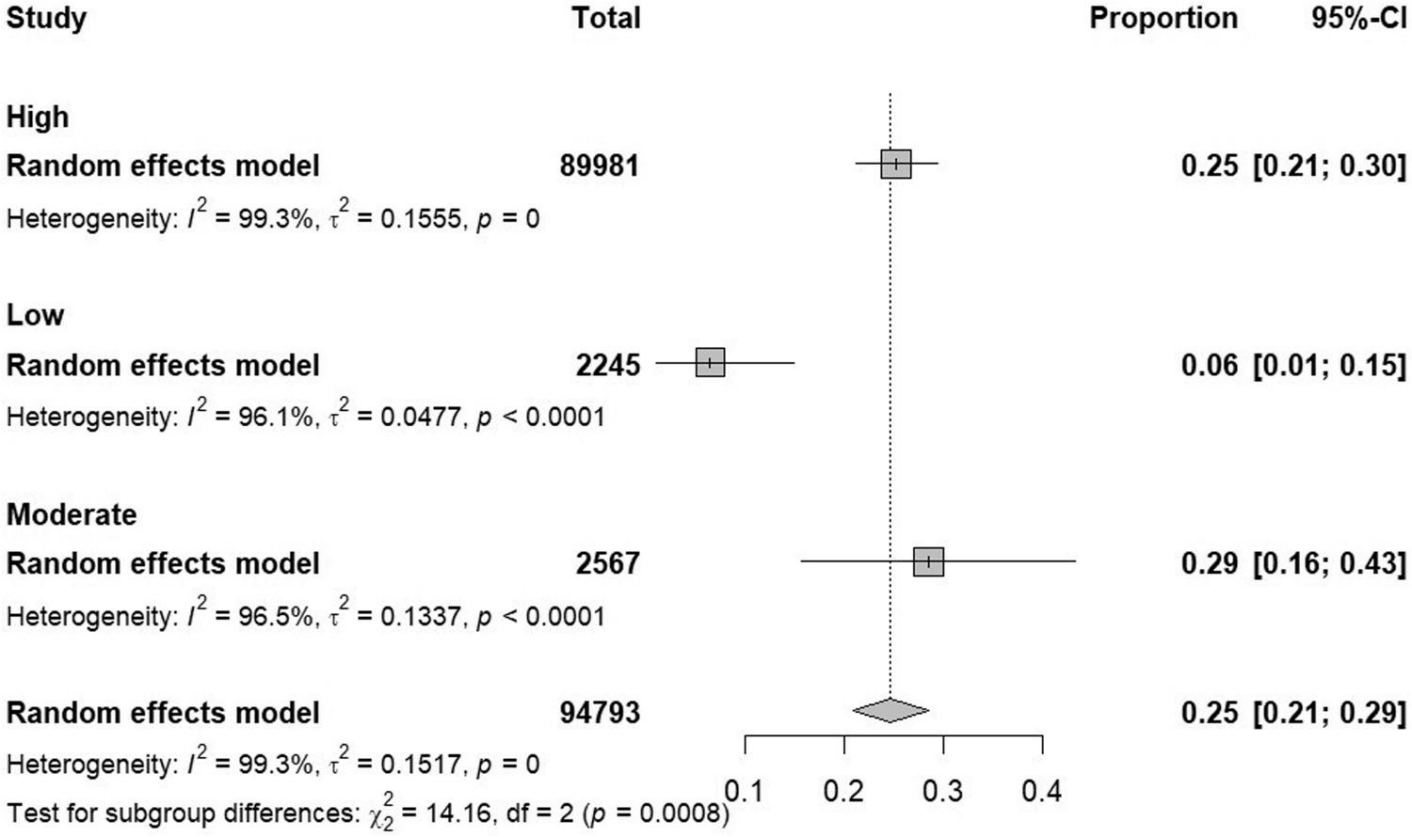
Subgroup meta‐analysis by quality score for vancomycin‐resistant *E. faecium* prevalence.

### 3.7 Publication Bias and Sensitivity Analysis

We evaluated publication bias using a funnel plot (Figure 25). Egger's regression test indicated significant publication bias (*p* < 0.001). After adjustment using the trim‐and‐fill method, the pooled prevalence was 33% (95% CI: 28%–39%) (Figure 26).

**Figure 25:**
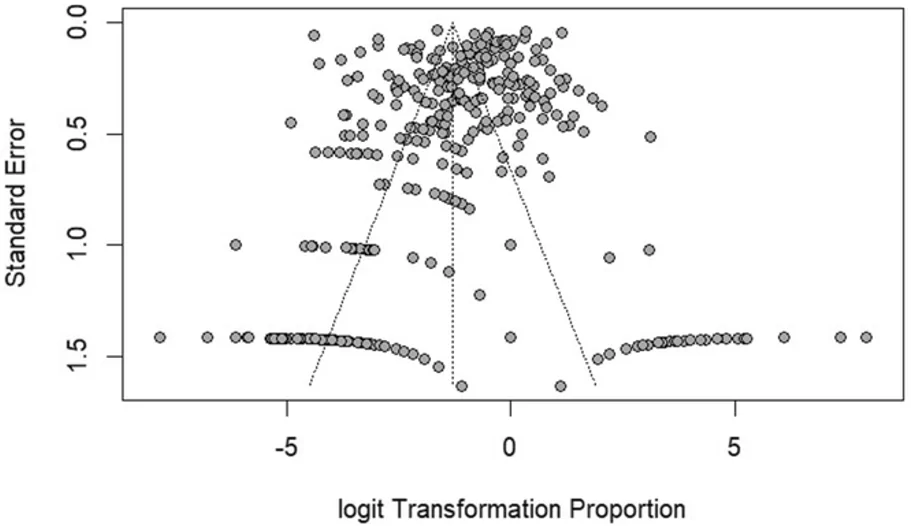
Funnel plot for assessment of publication bias in the included studies for vancomycin‐resistant *E. faecium* prevalence.

**Figure 26:**
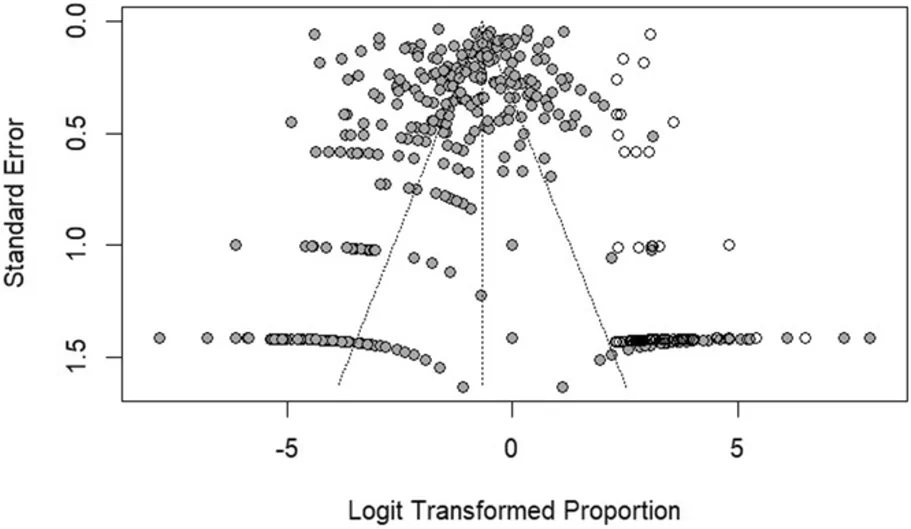
Trim‐and‐fill analysis to adjust for potential publication bias for vancomycin‐resistant *E. faecium* prevalence.

Sensitivity analysis using the leave‐one‐out approach showed that excluding any single study did not substantially affect the pooled prevalence estimate, confirming the robustness and stability of the overall findings.

## 4 Discussion

The rising prevalence of VRE isolates is considered a major threat to public health, particularly due to increasing rates of VREfm and VREfs infections. Therefore, this systematic review and meta‐analysis aimed to provide a comprehensive understanding of the proportion of VREfm and VREfs, and their temporal changes across 349 studies between 2000 and 2025.

Our meta‐analysis findings showed that the pooled prevalence of VREfm was higher than that of VREfs (25% vs. 5%). However, due to significant publication bias, we used the trim‐and‐fill method, and the pooled prevalence changed to 33% and 8% for VREfm and VREfs, respectively. Consistent with these findings, Shrestha et al. (Shrestha et al. 2021), in a meta‐analysis (up to 2020) of VRE clinical isolates from Asia, reported a higher prevalence of VREfm than of VREfs (22.40% vs. 3.70%). Similarly, Smout et al. (Smout et al. 2023), in another meta‐analysis of VRE clinical isolates from India, showed a higher prevalence of VREfm than of VREfs (58.15% vs. 36.41%). In contrast, a meta‐analysis (up to 2016) of VRE clinical isolates from Iran by Emaneini et al. (Emaneini et al. 2016) showed a higher prevalence of VREfs than VREfm. The predominance of VREfm in our analysis and in most other meta‐analyses may be explained by the increased adaptation of healthcare‐associated *E. faecium* lineages to the hospital environment and their ability to acquire antimicrobial resistance determinants (Zhou et al. 2020b).

In this meta‐analysis, we found a significant increase in the prevalence of VREfs and VREfm over the last two decades, from 1% before 2000 to 12% during 2021‐2025 for VREfs and from 11% before 2000 to 29% during 2021‐2025 for VREfm.

Consistent with our findings, time‐related increases in VRE prevalence have also been described in some previous meta‐analyses (Jabbari Shiadeh et al. 2019; Smout et al. 2023). Jabbari Shiadeh et al. (Jabbari Shiadeh et al. 2019) in the meta‐analysis of VRE blood isolates showed an increase in the prevalence of VREfm from 8% during 2000–2005 to 22% during 2010‐2016. They also indicated that the prevalence of VREfs increased from 1% to 2.7% during this period. Smout et al. in a meta‐analysis (Smout et al. 2023) in India up to 2020 showed a three‐fold increase in VRE prevalence from 4.8% between 2000 and 2010 to 14.1% between 2011 and 2020; also, a meta‐analysis by Huang et al. (Huang et al. 2025) revealed an increase in the prevalence of VREfm from 13.3% between 2000 and 2019 to 17.5% between 2020 and 2022. The findings indicate that the increase in VRE is not limited to a single geographic area or to a particular studied population. Continued antimicrobial selective pressure and the persistence and spread of healthcare‐adapted VRE lineages could explain the trend; however, differences in surveillance intensity and improvements in detection methods may also explain the higher VRE estimates in recent periods.

In this meta‐analysis, the highest prevalence of VREfs and VREfm was found in Africa (13%) and South America (57%), respectively, whereas the lowest prevalence of VREfs and VREfm was observed in Oceania (0%) and Africa (5%), respectively. Likewise, Jabbari et al. (Jabbari Shiadeh et al. 2019), based on the WHO regional categorization, reported the highest estimates of VREfs in the Eastern Mediterranean (5%) and the Americas (4.5%), while the lowest prevalence was found in the Western Pacific (0.6%). In addition, these investigators reported that the Eastern Mediterranean (23.2%) and Western Pacific (20.5%) had the highest prevalence of VREfm, while South‐East Asia (6%) and Europe (6.5%) had the lowest prevalence. Huang et al. (Huang et al. 2025) reported the highest prevalence of VREfm clinical isolates in the Western Pacific region (23.1%), whereas the lowest was in Europe (9.3%).

The country subgroup analysis for VREfs showed that Greece (50%) and Slovakia (44%) had the highest pooled prevalence. Similarly, the country subgroup analysis for VREfm showed that Ireland (100%; *n* = 39) and Argentina (74%; *n* = 46) had the highest pooled prevalence rates. However, these proportions were calculated based on a relatively small number of isolates. Also, in some countries, namely Greece, Slovakia, and Argentina, significant heterogeneity was observed. Therefore, more studies with larger sample sizes are essential to provide reliable prevalence estimates. Several meta‐analyses also evaluated subgroup analysis by country or region. Alemayehu et al. (Alemayehu and Hailemariam 2020), in a meta‐analysis on the African continent, reported that South Africa had the highest VRE prevalence (74.8%). Also, Shrestha et al. (Shrestha et al. 2021), in a meta‐analysis in the Asian continent, showed that Western Asian countries, including Iran, Saudi Arabia, Jordan, and Kuwait, had the highest prevalence of VRE, with an average estimate of 11.4%. This variation in prevalence estimates between our data and those of other meta‐analyses may reflect differences in antimicrobial exposure, healthcare infrastructure, infection prevention and control practices, the circulation of healthcare‐adapted VRE lineages, and the availability and intensity of surveillance across various regions (Cimen et al. 2023).

In contrast, our meta‐analysis showed near‐zero pooled prevalences of VREfm in Uganda (*n* = 46), Tunisia (*n* = 292), and Norway (*n* = 150), and of VREfs in South Korea and Saudi Arabia.

The heterogeneity of zero (I^2^ = 0%) across these countries indicates high consistency among the included studies, with VRE occurrence being zero or very low. In contrast to our meta‐analysis, Alemayehu et al. (Alemayehu and Hailemariam 2020), in a meta‐analysis conducted in Africa, reported VRE prevalence rates of 9.8% in Uganda and 6.5% in Tunisia, revealing some differences between our findings and those of their studies. This discrepancy may be due to several factors, including differences in study periods, sample sizes, sample sources (clinical or environmental), and the populations studied. In addition, discrepancies in antimicrobial surveillance programs and antibiotic consumption patterns across countries may affect VRE rates.

Subgroup analysis by isolate origin showed that most VREfs isolates were isolated from clinical samples rather than non‐clinical sources. However, the pooled prevalence of vancomycin resistance was lower in clinical samples than in non‐clinical sources (4% vs. 8%). Moreover, VREfs was highly prevalent in some non‐clinical sources, such as environmental water (26%) and animal stool (40%). These findings suggest that these two non‐clinical sources may serve as important environmental reservoirs and play a critical role in the circulation and spread of VRE clones. Also, *E. faecalis* of animal origin appears to pose a risk to human health because the same types of *E. faecalis* isolated from animals, meat, and fecal samples have also been detected in human samples from the community and in patients with bloodstream infections (Hammerum 2012). Furthermore, increased resistance among non‐clinical sources could suggest an underestimation of the environmental impact of VRE in surveillance that focuses primarily on clinical isolates. However, this conclusion is based on a small proportion of total isolates derived from non‐clinical sources. Overall, this finding points to the need to include more non‐clinical data in VRE surveillance, especially in settings associated with transmission by animals and water, which may contribute to the persistence and spread of resistant enterococci.

For VREfm isolates, subgroup analysis by isolate origin revealed the highest prevalence in rectal swabs (76%) and hospital environmental samples (74%). This finding may reflect the capacity for high colonization of VREfm in the gastrointestinal tract and its long‐term persistence in healthcare settings, which in turn may facilitate VREfm transmission among hospitalized patients. In parallel to our findings, Kramer et al., in a systematic review, found that *Enterococcus* spp. can survive on hospital inanimate surfaces for 5 days to 4 months (Kramer et al. 2006). Furthermore, the comparable prevalence of VREfm in rectal swabs and the hospital environmental samples may reflect the role of colonized patients and contaminated surfaces in sustaining VRE circulation in healthcare settings (Arias and Murray 2012; Donskey 2004).

Subgroup analysis by infection setting showed that VREfm prevalence was higher in hospital‐acquired infections than in community‐acquired infections (5% vs. 3%), but the difference was not significant (*p* > 0.05). In contrast, the prevalence of VREfm was significantly (*p* < 0.05) higher in hospital‐acquired infections than in community‐acquired infections (29% vs. 1%). However, the studies included in this subgroup showed high heterogeneity. The higher prevalence of VRE in hospital‐acquired infections may be attributed to the selective pressure of antibiotics, along with long‐term hospitalization, ICU stay, and extensive use of invasive medical devices, which may result in a higher chance of VRE transmission and infection in nosocomial settings (Mutters et al. 2013; Monteserin and Larson 2016).

Subgroup analysis by diagnostic methods demonstrated that studies using biochemical tests (culture‐based) combined with multiplex PCR or PCR had the highest proportions for diagnosing both VREfs and VREfm, suggesting that combining phenotypic and molecular methods may improve diagnostic accuracy. However, PCR alone for *E. faecalis* and API Strep for VREfm showed the lowest prevalence rates. Our results are partially consistent with findings of Alemayehu et al. (Alemayehu and Hailemariam 2020) meta‐analysis, which reported higher VRE prevalence in studies using PCR than in those using biochemical methods. These discrepancies across studies may be due to differences in methods used for species identification and resistance detection. Overall, combined phenotypic and molecular approaches seem to improve VRE detection. However, culture‐based methods alone may fail to detect VREs because of limitations in species identification or detection of resistance determinants.

In the subgroup analysis of AST methods, studies that used disc diffusion combined with E‐test methods had the highest prevalence (21%) of VREfs, and broth microdilution combined with E‐test methods had the lowest prevalence (0‐1%). Also, studies using the E‑test reported the highest prevalence (64%) of VREfm, followed by studies employing disc diffusion combined with E‐test methods (35%). Overall, most of the VREfs and VREfm isolates included in this meta‐analysis were identified as vancomycin‐resistant using the disk diffusion method. However, the high heterogeneity among the studies included in this subgroup for several AST methods reflects the different sensitivities of the techniques used to determine vancomycin resistance. Similarly, Shrestha et al. (Zaheer et al. 2020), in a meta‐analysis, reported that disk diffusion was the most common AST method for vancomycin resistance, with a pooled prevalence of 8.7%, followed by studies using the E‐test, with a similar prevalence of 8.3%. These findings revealed that disk diffusion and E‐test are commonly used AST methods for detecting vancomycin resistance, although the reported prevalence estimates varied across methods and study populations.

Subgroup analysis of AST guidelines showed that studies using CLSI criteria for VREfs and EUCAST criteria for VREfm had the highest pooled prevalence rates. These two guidelines included the largest number of isolates; however, studies showed high heterogeneity. Consistent with our meta‐analysis, Huang et al (Dolna et al. 2008). reported that most *E. faecium* isolates were interpreted as vancomycin‐resistant based on CLSI, with a pooled prevalence of 4.5%. In contrast, studies using EUCAST had a higher prevalence of vancomycin resistance, at 14%. These results align with our observation that the prevalence of vancomycin resistance may be influenced by the AST guidelines used and may reflect differences in breakpoint values and susceptibility categorization between CLSI and EUCAST.

This meta‐analysis has several limitations. First, there was substantial heterogeneity across the studies included in this meta‐analysis, despite the use of different subgroup analyses. Second, the included studies differed substantially in AST methods, bacterial identification techniques, and AST guidelines, which may have influenced the pooled prevalence rates. Third, data availability was substantially inconsistent across countries and continents; therefore, some pooled prevalence estimates were based on a limited number of studies or isolates. Fourth, because most included studies omitted important variables such as infection control measures, genes encoding vancomycin resistance, and clinical data on patients, we did not analyze these subgroups. Finally, the observational nature of the included studies limits causal inference about the global prevalence of VRE.

These limitations highlight several areas for future research. First, further studies are needed to standardize AST methods and interpretive criteria, as methodological differences may cause variability in reported VRE prevalence and limit comparability across studies. Internationally standardized surveillance frameworks, such as the WHO Global Antimicrobial Resistance and Use Surveillance System (GLASS), can improve the consistency, quality, and representativeness of antimicrobial resistance data across countries, particularly in regions with limited surveillance capacity (World Health Organization 2023). Second, molecular epidemiological approaches, including whole‐genome sequencing, should be incorporated to characterize vancomycin resistance determinants and distinguish clonal transmission from the dissemination of resistance genes. Recent genomic surveillance of VRE isolates has demonstrated that integrating genotypic data with routine phenotypic testing can identify cryptic resistance and reveal hospital‐associated transmission patterns that may otherwise remain undetected (Schrader et al. 2025). Finally, future multicenter prospective studies incorporating clinical, environmental, and animal sampling are also warranted to improve geographic representation and clarify transmission pathways across the One Health continuum. Such studies could provide more robust evidence for targeted infection prevention, antimicrobial stewardship, and VRE control strategies (Brodrick et al. 2016).

## 5 Conclusion

This systematic review and meta‐analysis globally indicated a significantly higher pooled prevalence of VREfm than VREfs. The prevalence of both species has increased significantly in the past two decades. The present study also provides a more complete insight into the global epidemiology of VRE than previous reviews, which focused mainly on clinical isolates, because it integrated data from clinical and environmental sources. The high prevalence of VREfm in rectal swabs and hospital environmental samples, and the increased prevalence of VREfs in environmental water and animal stool, suggest a role for environmental and healthcare‐associated reservoirs in the persistence and dissemination of VRE.

These findings support One Health surveillance strategies that integrate clinical, environmental, and animal surveillance to improve infection prevention, antimicrobial stewardship, and public health responses to VRE dissemination. However, the pooled estimates should be interpreted with caution because of the large heterogeneity across studies and uneven geographic representation.

The observed global trends demonstrated an increasing burden of vancomycin resistance. However, differences in pooled prevalence estimates of AST methods and interpretive guidelines further emphasize the need for standardized laboratory methods to detect and monitor VRE accurately. Hence, future studies should include standardized AST methods, genomic characterization of resistance determinants, and longitudinal surveillance in clinical and environmental settings to understand transmission pathways better and guide targeted control interventions.

## Author Contributions

**Negar Narimisa:** formal analysis, software. **Tahereh Navidifar:** writing – original draft, writing – review and editing. **Tuqa T. Hameed:** conceptualization, investigation. **Roghayeh Mohammadzadeh:** investigation. **Faranak Abdoli:** investigation. **Mahsa Ghamari:** investigation. **Delaram Nourollahi:** investigation. **Fatemeh Ansari:** investigation. **Arefeh Ansari:** investigation. **Narjess Bostanghadiri:** writing – review and editing, supervision, project administration.

## Funding

The authors have nothing to report.

## Ethics Statement

The study was approved by the Ethics Committee of Tehran University of Medical Sciences (approval No. IR.TUMS.SPH.REC.1404.245).

## Conflicts of Interest

The authors declare no conflicts of interest.

## Supporting information


Supporting File 1

Supporting File 2

Supporting File 3

Supporting File 4

Supporting File 5

## Data Availability

The data that support the findings of this study are available in the Supporting material of this article.
