# Supplementary material for: Global Prevalence of Vancomycin‐Resistant Enterococcus faecium and Enterococcus faecalis Isolated From Clinical and Environmental Sources: A Systematic Review and Meta‐Analysis
Source: Microbiologyopen. 2026 Oct 13;15(5):e70434. doi: 10.1002/mbo3.70434 (PMC13662538; doi:10.1002/mbo3.70434)
Supplement: Supplementary file 1 [file MBO3-15-e70434-s002.pdf]

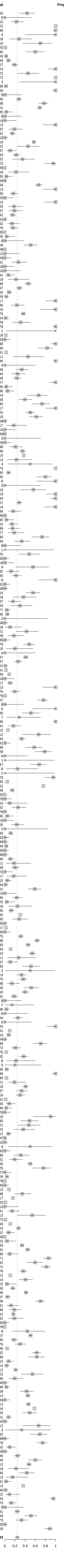

Study	Events	Total	Proportion	95%−CI	Weight
Bhatt,2015	22	50	0.44	[0.30; 0.59]	0.3%
David Patrick,2013	0	5	0.00	[0.00; 0.52]	0.2%
Dalia Moemen,2015	12	52	0.23	[0.13; 0.37]	0.3%
Kelvin W. C. Leong,2018	222	222	1.00	[0.98; 1.00]	0.3%
Eichel,2022	96	96	1.00	[0.96; 1.00]	0.3%
R.B. Ibrahim,2011	4	4	1.00	[0.40; 1.00]	0.2%
Kumar Das,2020	5	18	0.28	[0.10; 0.53]	0.3%
Fakhri Haghi,2019	7	10	0.70	[0.35; 0.93]	0.3%
Lin Huang,2019	324	26620	0.01	[0.01; 0.01]	0.3%
Fahimeh Hajiahmadi,2016	18	30	0.60	[0.41; 0.77]	0.3%
Freitas,2018	4	165	0.02	[0.01; 0.06]	0.3%
Billington,2014	12	169	0.07	[0.04; 0.12]	0.3%
Marzieh Aligholi,2008	30	157	0.19	[0.13; 0.26]	0.3%
Li Gu,2009	23	23	1.00	[0.85; 1.00]	0.3%
Teresa M. Braga,2012	10	22	0.45	[0.24; 0.68]	0.3%
Lee,2022	1372	1372	1.00	[1.00; 1.00]	0.3%
W. Grzybowska,2009	3	3	1.00	[0.29; 1.00]	0.2%
Bruna C. Gomes,2008	3	139	0.02	[0.00; 0.06]	0.3%
Lytsy,2017	60	60	1.00	[0.94; 1.00]	0.3%
Yohannes E. Messele,2022	0	9	0.00	[0.00; 0.34]	0.3%
Lo,2011	95	345	0.28	[0.23; 0.33]	0.3%
Mehdi Mirzaii,2023	68	88	0.77	[0.67; 0.86]	0.3%
Kuo,2018	378	551	0.69	[0.65; 0.72]	0.3%
Hanna Rózanska,2019	1	35	0.03	[0.00; 0.15]	0.3%
Birri,2012	0	9	0.00	[0.00; 0.34]	0.3%
Assadian,2002	0	14	0.00	[0.00; 0.23]	0.3%
Lee,2016	13	13	1.00	[0.75; 1.00]	0.3%
Luca Busani,2004	7	37	0.19	[0.08; 0.35]	0.3%
Lu,2024	21	142	0.15	[0.09; 0.22]	0.3%
Maria Cristina Ossiprandi,2006	0	52	0.00	[0.00; 0.07]	0.3%
Francis F. Arhin,2009	469	819	0.57	[0.54; 0.61]	0.3%
Dowzicky,2019	10	22	0.45	[0.24; 0.68]	0.3%
Dowzicky,2019	5	47	0.11	[0.04; 0.23]	0.3%
Dowzicky,2019	30	133	0.23	[0.16; 0.31]	0.3%
Dowzicky,2019	7	20	0.35	[0.15; 0.59]	0.3%
Luciana Furlaneto−Maia,2014	22	23	0.96	[0.78; 1.00]	0.3%
Jannati,2020	0	235	0.00	[0.00; 0.02]	0.3%
Zahraa Nafeh Aziz,2025	4	18	0.22	[0.06; 0.48]	0.3%
López−Luis,2021	1	33	0.03	[0.00; 0.16]	0.3%
Nam Hee Kim,2020	0	106	0.00	[0.00; 0.03]	0.3%
López−Luis,2021	106	159	0.67	[0.59; 0.74]	0.3%
Boisivon,1997	13	13	1.00	[0.75; 1.00]	0.3%
Ahmed Mhawesh,2022	17	100	0.17	[0.10; 0.26]	0.3%
Ngbede,2023	0	20	0.00	[0.00; 0.17]	0.3%
Al−Mohri,2007	15	15	1.00	[0.78; 1.00]	0.3%
Mehmet Butcu,2011	6	33	0.18	[0.07; 0.35]	0.3%
Ernesto Calderón−Jaimes,2003	7	37	0.19	[0.08; 0.35]	0.3%
Adam,2011	22	142	0.15	[0.10; 0.23]	0.3%
Asadian,2016	0	91	0.00	[0.00; 0.04]	0.3%
Varun Goel,2016	6	42	0.14	[0.05; 0.29]	0.3%
Arshadi,2017	13	79	0.16	[0.09; 0.26]	0.3%
Abbassi,2009	0	235	0.00	[0.00; 0.02]	0.3%
Malik,2022	1	26	0.04	[0.00; 0.20]	0.3%
Ahmed,2014	0	8	0.00	[0.00; 0.37]	0.3%
SULE CELIK,2014	30	59	0.51	[0.37; 0.64]	0.3%
Abat,2015	1	469	0.00	[0.00; 0.01]	0.3%
Bell,2003	0	11	0.00	[0.00; 0.28]	0.3%
Bell,2003	0	28	0.00	[0.00; 0.12]	0.3%
Bell,2003	11	41	0.27	[0.14; 0.43]	0.3%
Bell,2003	0	18	0.00	[0.00; 0.19]	0.3%
Bell,2003	0	10	0.00	[0.00; 0.31]	0.3%
Bell,2003	3	41	0.07	[0.02; 0.20]	0.3%
Bogut,2021	4	18	0.22	[0.06; 0.48]	0.3%
Mansour Goudarzi,2018	87	188	0.46	[0.39; 0.54]	0.3%
Gümüs,2023	68	237	0.29	[0.23; 0.35]	0.3%
Banerjee,2015	13	155	0.08	[0.05; 0.14]	0.3%
Naidoo,2023	3	109	0.03	[0.01; 0.08]	0.3%
Katharine Bar,2006	17	17	1.00	[0.80; 1.00]	0.3%
Deharya,2024	11	46	0.24	[0.13; 0.39]	0.3%
Freitas,2006	10	10	1.00	[0.69; 1.00]	0.3%
Geoffrey W. Coombs,2014	137	375	0.37	[0.32; 0.42]	0.3%
Nur Nashyirah,2023	1	64	0.02	[0.00; 0.08]	0.3%
Carlos Bantar,2000	9	29	0.31	[0.15; 0.51]	0.3%
Morris,1995	78	78	1.00	[0.95; 1.00]	0.3%
Susana Correia,2013	1	1	1.00	[0.03; 1.00]	0.1%
Pan,2024	36	1618	0.02	[0.02; 0.03]	0.3%
Kapoor,2005	0	33	0.00	[0.00; 0.11]	0.3%
Papadimitriou−Olivgeris,2016	38	38	1.00	[0.91; 1.00]	0.3%
Diana Brlek Gorski,2024	25	30	0.83	[0.65; 0.94]	0.3%
Kowalska−Krochmal,2008	15	591	0.03	[0.01; 0.04]	0.3%
Hitkova,2019	5	11	0.45	[0.17; 0.77]	0.3%
Saengsuwan,2021	90	90	1.00	[0.96; 1.00]	0.3%
Kouidhi,2011	0	4	0.00	[0.00; 0.60]	0.2%
Sung−Yeon Cho,2013	22	66	0.33	[0.22; 0.46]	0.3%
Maria Cecilia Di Pentima,2014	11	44	0.25	[0.13; 0.40]	0.3%
Hota,2024	26	109	0.24	[0.16; 0.33]	0.3%
Jozef Kuzma,2022	27	27	1.00	[0.87; 1.00]	0.3%
Masoud Alebouyeh,2005	1	35	0.03	[0.00; 0.15]	0.3%
BAHRAM FATHOLAHZADEH,2006	2	34	0.06	[0.01; 0.19]	0.3%
Robert Ducu,2019	16	24	0.67	[0.45; 0.84]	0.3%
Pamela Alatorre−Fernández MD,2016	23	58	0.40	[0.27; 0.53]	0.3%
Canan Kuzdan,2014	22	30	0.73	[0.54; 0.88]	0.3%
A. M. LITTVIK,2006	17	17	1.00	[0.80; 1.00]	0.3%
Sung−Yeon Cho, M.D,2018	60	120	0.50	[0.41; 0.59]	0.3%
Fawzia E Alotaibi,2017	33	53	0.62	[0.48; 0.75]	0.3%
Ramonalseppi,2020	0	68	0.00	[0.00; 0.05]	0.3%
Beata Dolka,2020	3	17	0.18	[0.04; 0.43]	0.3%
Kamonnaree Chotinantakul,2017	0	15	0.00	[0.00; 0.22]	0.3%
Grebstad Rabbi Amuasi−Felicia A. Owusu,2023	0	89	0.00	[0.00; 0.04]	0.3%
Aziz Bouymajane,2018	0	3	0.00	[0.00; 0.71]	0.2%
Donado−Godoy,2015	0	26	0.00	[0.00; 0.13]	0.3%
Saba Asgharzadeh,2021	10	48	0.21	[0.10; 0.35]	0.3%
Arockia Doss Susai backiam,2023	86	246	0.35	[0.29; 0.41]	0.3%
Sung Young Lee,2024	792	2115	0.37	[0.35; 0.40]	0.3%
Setareh Mamishi,2020	3	13	0.23	[0.05; 0.54]	0.3%
M. Falcone,2019	2	4	0.50	[0.07; 0.93]	0.2%
Jan Liese,2019	773	773	1.00	[1.00; 1.00]	0.3%
Keryn J Christiansen,2007	13	180	0.07	[0.04; 0.12]	0.3%
Sanja Jakovac,2016	28	35	0.80	[0.63; 0.92]	0.3%
Acácio Salamandane,2023	8	8	1.00	[0.63; 1.00]	0.3%
Manuela Oliveira,2016	0	3	0.00	[0.00; 0.71]	0.2%
Esra Seker,2023	9	16	0.56	[0.30; 0.80]	0.3%
Egan,2020	19	19	1.00	[0.82; 1.00]	0.3%
Balázs Libisch,2009	34	34	1.00	[0.90; 1.00]	0.3%
Michael Brandon,2013	75	267	0.28	[0.23; 0.34]	0.3%
Guerrero−Ramos,2016	14	14	1.00	[0.77; 1.00]	0.3%
Solayide A. Adesida,2017	6	36	0.17	[0.06; 0.33]	0.3%
SR Moaddab,2004	0	85	0.00	[0.00; 0.04]	0.3%
Min Liu,2023	1	30	0.03	[0.00; 0.17]	0.3%
Farooq Ahmad,2024	9	62	0.15	[0.07; 0.26]	0.3%
Katarzyna Grudlewska,2023	6	39	0.15	[0.06; 0.31]	0.3%
MM Salem−Bekhit,2011	5	27	0.19	[0.06; 0.38]	0.3%
Canan Kuzdan1,2014	22	30	0.73	[0.54; 0.88]	0.3%
Giuseppe Todeschin,2005	13	39	0.33	[0.19; 0.50]	0.3%
Ricardo Titze−de,2004	0	9	0.00	[0.00; 0.34]	0.3%
Jinglu Sun−Xiaobo,2009	0	1	0.00	[0.00; 0.97]	0.1%
Mehmet Elmali,2018	12	25	0.48	[0.28; 0.69]	0.3%
Aylin Kasimoglu−Dogru,2010	0	40	0.00	[0.00; 0.09]	0.3%
Houssem Ben Yahia,2018	0	19	0.00	[0.00; 0.18]	0.3%
E. Seker,2023	5	9	0.56	[0.21; 0.86]	0.3%
Edet E. Udo− Noura,2002	4	32	0.12	[0.04; 0.29]	0.3%
Malihe Talebi,2008	19	85	0.22	[0.14; 0.33]	0.3%
Veronika Oravcova,2013	9	9	1.00	[0.66; 1.00]	0.3%
S. Sreeja,2012	0	31	0.00	[0.00; 0.11]	0.3%
Jéssica Lopes−Teresa Conceição,2024	0	58	0.00	[0.00; 0.06]	0.3%
Hyeah Choi,2022	30	54	0.56	[0.41; 0.69]	0.3%
Jae−Cheol Kwon,2012	7	19	0.37	[0.16; 0.62]	0.3%
Ayan Kumar,2022	6	37	0.16	[0.06; 0.32]	0.3%
Ayan Kumar Das−Mridu Dudeja,2022	6	20	0.30	[0.12; 0.54]	0.3%
Nicholas Figueiredo−Hajer,2009	3	77	0.04	[0.01; 0.11]	0.3%
Min−Chan Kim,2017	9	185	0.05	[0.02; 0.09]	0.3%
Samiah Hamad S Al−Mijalli,2022	0	2	0.00	[0.00; 0.84]	0.2%
Sarika Jain,2011	0	58	0.00	[0.00; 0.06]	0.3%
Behice Kurtaran, MD,2011	2	19	0.11	[0.01; 0.33]	0.3%
Hesam Alizade,2022	9	21	0.43	[0.22; 0.66]	0.3%
Aslantas,2019	5	28	0.18	[0.06; 0.37]	0.3%
Maureen Boost,2004	0	35	0.00	[0.00; 0.10]	0.3%
Arshadi,2017	38	79	0.48	[0.37; 0.60]	0.3%
Onaran,2019	2	10	0.20	[0.03; 0.56]	0.3%
David Patrick Kateete,2013	0	4	0.00	[0.00; 0.60]	0.2%
Ali Al Bshabshe,2024	143	347	0.41	[0.36; 0.47]	0.3%
Andrew J. Denisuik,2018	36	137	0.26	[0.19; 0.34]	0.3%
CARINA CLAESSON,2007	1	101	0.01	[0.00; 0.05]	0.3%
CARINA CLAESSON−LENNART,2008	6	241	0.02	[0.01; 0.05]	0.3%
S.K. Bouchillon−B.M.,2004	83	949	0.09	[0.07; 0.11]	0.3%
Nadjette Bourafa−Ce,2016	0	18	0.00	[0.00; 0.19]	0.3%
Douglas J. Biedenbach,2007	12	117	0.10	[0.05; 0.17]	0.3%
Ali M. Somily,2016	13	13	1.00	[0.75; 1.00]	0.3%
Techilo Habtemariam,2024	15	76	0.20	[0.11; 0.30]	0.3%
Wei Jia,2014	5	679	0.01	[0.00; 0.02]	0.3%
Mona NASAJ,2015	51	67	0.76	[0.64; 0.86]	0.3%
Hasan Ejaz,2019	0	8	0.00	[0.00; 0.37]	0.3%
Biggel,2021	6	6	1.00	[0.54; 1.00]	0.2%
Ali Jahansepas,2017	18	35	0.51	[0.34; 0.69]	0.3%
Ashfaq Ahmad Shah,2022	2	7	0.29	[0.04; 0.71]	0.2%
S. Ameri,2008	96	96	1.00	[0.96; 1.00]	0.3%
Aasish Karna,2019	7	41	0.17	[0.07; 0.32]	0.3%
Malihe Talebi,2008	19	315	0.06	[0.04; 0.09]	0.3%
Eman A. El−Masry,2015	8	12	0.67	[0.35; 0.90]	0.3%
Folasade Muibat Adeyemi,2021	61	184	0.33	[0.26; 0.40]	0.3%
M. L. FERNA´ NDEZ−GUERRERO,2002	0	31	0.00	[0.00; 0.11]	0.3%
Toshiki Kajihara,2015	22	38	0.58	[0.41; 0.74]	0.3%
Daniely M. Silva,2017	22	28	0.79	[0.59; 0.92]	0.3%
Y.−J. Kim,2018	0	6	0.00	[0.00; 0.46]	0.2%
Se−Hyung Kim,2020	0	1	0.00	[0.00; 0.97]	0.1%
Youn Jeong Kima,2019	25	37	0.68	[0.50; 0.82]	0.3%
M. Kierzkowska,2014	2	8	0.25	[0.03; 0.65]	0.3%
Erick Odoyo,2023	0	1	0.00	[0.00; 0.97]	0.1%
I.C.V. Palazzo,2011	22	23	0.96	[0.78; 1.00]	0.3%
S.G.K. Hansen,2024	105	2135	0.05	[0.04; 0.06]	0.3%
Helio S. Sader,2011	1867	2462	0.76	[0.74; 0.78]	0.3%
Dokyun Kim,2024	45	308	0.15	[0.11; 0.19]	0.3%
Karimaei,2016	0	432	0.00	[0.00; 0.01]	0.3%
Birgul Kacmaz,2005	0	25	0.00	[0.00; 0.14]	0.3%
Saraswathy Palanisamy,2013	1	10	0.10	[0.00; 0.45]	0.3%
M.G. Karmarkar,2004	11	42	0.26	[0.14; 0.42]	0.3%
J. Gray,1994	0	76	0.00	[0.00; 0.05]	0.3%
Abdulhakim Abamecha,2015	2	40	0.05	[0.01; 0.17]	0.3%
Wenwei Ouyang,2016	0	19	0.00	[0.00; 0.18]	0.3%
Imane Hannaoui,2016	13	55	0.24	[0.13; 0.37]	0.3%
Hamid Heidari,2016	0	2	0.00	[0.00; 0.84]	0.2%
R.N. Gruneberg,1998	21	183	0.11	[0.07; 0.17]	0.3%
Iseppi,2015	0	42	0.00	[0.00; 0.08]	0.3%
N. KOBAYASHI,2000	0	46	0.00	[0.00; 0.08]	0.3%
Stig Harthug,2000	4	149	0.03	[0.01; 0.07]	0.3%
PO−REN HSUEH,2001	0	45	0.00	[0.00; 0.08]	0.3%
YUTAKA NISHIMOTO,2005	0	46	0.00	[0.00; 0.08]	0.3%
Franz−Josef Schmitz,1999	10	90	0.11	[0.05; 0.19]	0.3%
Kere Kobayashi,2000	2	11	0.18	[0.02; 0.52]	0.3%
C. J. Henwood,2000	22	108	0.20	[0.13; 0.29]	0.3%
P A Mackowiak,1989	0	22	0.00	[0.00; 0.15]	0.3%
Anne Kjerulf,1996	3	17	0.18	[0.04; 0.43]	0.3%
RIKKE LYKKE POULSEN,1999	3	62	0.05	[0.01; 0.13]	0.3%
M. LINDSAY GRAYSON,1991	1	84	0.01	[0.00; 0.06]	0.3%
Benjamin P. Howden,2013	36	61	0.59	[0.46; 0.71]	0.3%
Tariq AA Madani,1999	0	15	0.00	[0.00; 0.22]	0.3%
G. NICOLETTI,2006	20	85	0.24	[0.15; 0.34]	0.3%
Arata Hidano,2015	1	25	0.04	[0.00; 0.20]	0.3%
Gary A. Noskin,1995	4	16	0.25	[0.07; 0.52]	0.3%
Heike von Baum,1999	18	145	0.12	[0.08; 0.19]	0.3%
Daniel Hornuss,2024	567	1845	0.31	[0.29; 0.33]	0.3%
Athraa Harjan Mohsen Aldulhaidhawi,2022	10	35	0.29	[0.15; 0.46]	0.3%
Y. HIRAKATA,1997	0	80	0.00	[0.00; 0.05]	0.3%
Gülsen HAZIROLAN,2020	6	257	0.02	[0.01; 0.05]	0.3%
Markus Hufnagel,2007	0	30	0.00	[0.00; 0.12]	0.3%
Coombs,GW,2014	118	375	0.31	[0.27; 0.36]	0.3%
Bahram Nikmanesh,2024	183	288	0.64	[0.58; 0.69]	0.3%
Geoffrey W Coombs,2016	175	380	0.46	[0.41; 0.51]	0.3%
Javier Aznar,2012	103	893	0.12	[0.10; 0.14]	0.3%
Maniya Arshadi,2019	88	163	0.54	[0.46; 0.62]	0.3%
Ümmühan Çay,2022	3	11	0.27	[0.06; 0.61]	0.3%
Priyanka Paul Biswas,2013	4	36	0.11	[0.03; 0.26]	0.3%
Ali Jahansepas,2019	18	35	0.51	[0.34; 0.69]	0.3%
Smeeta Huidrom,2016	1	2	0.50	[0.01; 0.99]	0.2%
A. Japoni,2009	37	113	0.33	[0.24; 0.42]	0.3%
Geoffrey W Coombs,2013	62	170	0.36	[0.29; 0.44]	0.3%
A. Lambiase,2007	5	36	0.14	[0.05; 0.29]	0.3%
Meharunisa Indikar,2024	26	50	0.52	[0.37; 0.66]	0.3%
Wui Wei,2014	60	150	0.40	[0.32; 0.48]	0.3%
M. Kresken,2009	26	142	0.18	[0.12; 0.26]	0.3%
Jaafar Rezvani,2016	0	9	0.00	[0.00; 0.34]	0.3%
Sangjoon Ryu,2025	1	7	0.14	[0.00; 0.58]	0.2%
X. Bertrand,2000	3	96	0.03	[0.01; 0.09]	0.3%
K. Gopalkrishna Bhat,1997	0	6	0.00	[0.00; 0.46]	0.2%
Snežana Brkić,2017	27	104	0.26	[0.18; 0.35]	0.3%
David P. Kateete,2019	0	4	0.00	[0.00; 0.60]	0.2%
Egan,2020	20	20	1.00	[0.83; 1.00]	0.3%
Lucia Mališová,2021	77	791	0.10	[0.08; 0.12]	0.3%
Dilek BULUT,2024	16	210	0.08	[0.04; 0.12]	0.3%
Jesús Fortún,2002	0	104	0.00	[0.00; 0.03]	0.3%
Noah Hackney,2019	34	48	0.71	[0.56; 0.83]	0.3%
Hanane Zerrouki,2021	15	72	0.21	[0.12; 0.32]	0.3%
Mariarosaria Boccella,2021	18	576	0.03	[0.02; 0.05]	0.3%
Alina Maria Borcan,2024	49	132	0.37	[0.29; 0.46]	0.3%
Anna Woz'niak−Biel,2019	2	9	0.22	[0.03; 0.60]	0.3%
Anna Woz'niak−Biel,2019	1	5	0.20	[0.01; 0.72]	0.2%
G. Pesavento,2014	5	140	0.04	[0.01; 0.08]	0.3%
Mohammad R. Pourshafie,2008	49	49	1.00	[0.93; 1.00]	0.3%
Wanqing Zhou,2020	58	1738	0.03	[0.03; 0.04]	0.3%
Amir Emamie,2023	4	21	0.19	[0.05; 0.42]	0.3%
Geoffrey W Coombs,2014	130	318	0.41	[0.35; 0.47]	0.3%
Hossein Samadi Kafil,2013	29	87	0.33	[0.24; 0.44]	0.3%
Jing−xian Yang,2024	35	116	0.30	[0.22; 0.39]	0.3%
Janet M. Manson,2004	3	181	0.02	[0.00; 0.05]	0.3%
Muhammad Arfat Yameen,2013	4	45	0.09	[0.02; 0.21]	0.3%
Nabil Ben Omar,2023	1	36	0.03	[0.00; 0.15]	0.3%
Xiaoyan Liao,2024	0	29	0.00	[0.00; 0.12]	0.3%
Davood Yadegarynia,2016	9	10	0.90	[0.55; 1.00]	0.3%
Natália Conceição,2011	17	35	0.49	[0.31; 0.66]	0.3%
Narges Dadfarmaa,2013	10	21	0.48	[0.26; 0.70]	0.3%
S. YASLIANI,2009	8	22	0.36	[0.17; 0.59]	0.3%
I. Gawryszewska,2016	8	112	0.07	[0.03; 0.14]	0.3%
Lynette M. Johnston,2004	0	97	0.00	[0.00; 0.04]	0.3%
Xiomara,2002	0	52	0.00	[0.00; 0.07]	0.3%
Félix Carrasco Calzada,2023	2	4	0.50	[0.07; 0.93]	0.2%
Félix Carrasco Calzada,2023	0	32	0.00	[0.00; 0.11]	0.3%
Tsering Yangzom,2020	12	38	0.32	[0.18; 0.49]	0.3%
Anita Williams,2024	8	41	0.20	[0.09; 0.35]	0.3%
Geoffrey W Coombs,2018	200	400	0.50	[0.45; 0.55]	0.3%
SUMRU ÇITAK,2004	19	25	0.76	[0.55; 0.91]	0.3%
Garima Yadav,2017	0	27	0.00	[0.00; 0.13]	0.3%
Ujjala Ghoshal,2006	10	229	0.04	[0.02; 0.08]	0.3%
A. BELICOVA,2007	0	178	0.00	[0.00; 0.02]	0.3%
P. Poeta,2005	0	45	0.00	[0.00; 0.08]	0.3%
Roberta Fontana,1998	77	913	0.08	[0.07; 0.10]	0.3%
Ghazal Zolfgar,2024	13	38	0.34	[0.20; 0.51]	0.3%
Kyungwon Lee−Sook−Jin Jang,2003	1027	6417	0.16	[0.15; 0.17]	0.3%
M. Pinholt,2013	3	243	0.01	[0.00; 0.04]	0.3%
N.N.I. Mastor,2021	0	20	0.00	[0.00; 0.17]	0.3%
Alina−Viorica Iancu,2023	6	41	0.15	[0.06; 0.29]	0.3%
Chimanjita Phukan,2016	7	13	0.54	[0.25; 0.81]	0.3%
Vanja Piezzi,2020	30	2173	0.01	[0.01; 0.02]	0.3%
SUK−KYUNG LIM,2006	61	556	0.11	[0.08; 0.14]	0.3%
Derek R J. Brown,2008	125	463	0.27	[0.23; 0.31]	0.3%
Trapti Sharma,2024	8	64	0.12	[0.06; 0.19]	0.3%
Eliot Hershberger,2005	0	1256	0.00	[0.00; 0.00]	0.3%
Mahmoud M. Tawfick,2020	1	23	0.04	[0.00; 0.22]	0.3%
Sarah V. Walker,2020	167	493	0.34	[0.30; 0.38]	0.3%
Geoffrey W Coombs,2020	221	491	0.45	[0.41; 0.50]	0.3%
Sachin M Darji,2024	3	10	0.30	[0.07; 0.65]	0.3%
Natkamon Saenhom,2022	61	71	0.86	[0.76; 0.93]	0.3%
Dan Alexandru Toc,2023	25	42	0.60	[0.43; 0.74]	0.3%
Fatemeh Shahi,2020	59	72	0.82	[0.71; 0.90]	0.3%
Zrinka Todoric,2022	64	176	0.36	[0.29; 0.44]	0.3%
A Tripathi,2016	46	421	0.11	[0.08; 0.14]	0.3%
Obaid Ullah,2015	22	54	0.41	[0.28; 0.55]	0.3%
WAVARE SANJAY M,2015	11	222	0.05	[0.03; 0.09]	0.3%
Qing−Yong Wang,2015	42	265	0.16	[0.12; 0.21]	0.3%
Geoffrey W Coombs,2020	249	596	0.42	[0.38; 0.46]	0.3%
Elham Jannatia,2023	5	97	0.05	[0.02; 0.12]	0.3%
Azin Sattari−Maraji,2019	76	108	0.70	[0.61; 0.79]	0.3%
Mohan Bilikallahalli,2023	5	41	0.12	[0.04; 0.26]	0.3%
Vishal C. Shete,2019	9	48	0.19	[0.09; 0.33]	0.3%
Jann−Tay Wang,2013	76	452	0.17	[0.13; 0.21]	0.3%
Heba Shehta Said,2019	6	32	0.19	[0.07; 0.36]	0.3%
Gülsah Tollu,2020	0	40	0.00	[0.00; 0.09]	0.3%
Moneoang−Modisane Simon,2009	0	176	0.00	[0.00; 0.02]	0.3%
Fatima Sana,2019	4	9	0.44	[0.14; 0.79]	0.3%
Geoffrey W Coombs,2024	334	657	0.51	[0.47; 0.55]	0.3%
Jatapat Hemapanpairoa,2021	26	145	0.18	[0.12; 0.25]	0.3%
Sanjeev Das,2021	23	76	0.30	[0.20; 0.42]	0.3%
Prakaithip Thongkoom,2012	199	4106	0.05	[0.04; 0.06]	0.3%
Mahnaz Saifi,2007	89	142	0.63	[0.54; 0.71]	0.3%
Shuan Tao,2022	24	38	0.63	[0.46; 0.78]	0.3%
Trapti Sharma,2025	4	52	0.08	[0.02; 0.19]	0.3%
Mallika Sengupta,2023	4	114	0.04	[0.01; 0.09]	0.3%
Stefania Stolfa,2021	110	512	0.21	[0.18; 0.25]	0.3%
Shahram Shahraki,2017	12	22	0.55	[0.32; 0.76]	0.3%
Nidhi Sood,2024	7	36	0.19	[0.08; 0.36]	0.3%
Vittoria Cinquepalmi,2012	0	45	0.00	[0.00; 0.08]	0.3%
Silvia Monteiro,2020	3	98	0.03	[0.01; 0.09]	0.3%
L. Adhikari,2016	25	58	0.43	[0.30; 0.57]	0.3%
Geoffrey W Coombs,2023	279	613	0.46	[0.42; 0.50]	0.3%
Leila Ben Said,2015	0	34	0.00	[0.00; 0.10]	0.3%
Geoffrey W Coombs,2023	285	613	0.46	[0.42; 0.51]	0.3%
Mei−Chun Lee,2021	1787	3054	0.59	[0.57; 0.60]	0.3%
Linda Labecka,2024	27	55	0.49	[0.35; 0.63]	0.3%
Zuzanna Kraszewska,2022	11	151	0.07	[0.04; 0.13]	0.3%
Robert S. Dungan,2021	0	55	0.00	[0.00; 0.06]	0.3%
Mona A. El−ZamkanD,2021	8	12	0.67	[0.35; 0.90]	0.3%
John C. Christenson,1998	1	3	0.33	[0.01; 0.91]	0.2%
M. Dan,1999	22	32	0.69	[0.50; 0.84]	0.3%
NASSIMA DJAHMI,2012	0	39	0.00	[0.00; 0.09]	0.3%
Barbara Araújo dos Santos,2020	58	78	0.74	[0.63; 0.84]	0.3%
F. MOHAMMADI,2015	5	52	0.10	[0.03; 0.21]	0.3%
STEVEN GORDON,1992	0	58	0.00	[0.00; 0.06]	0.3%
Christoph Lubbert,2015	46	184	0.25	[0.19; 0.32]	0.3%
Claudia Magoral−Tera n,2019	35	60	0.58	[0.45; 0.71]	0.3%
Geoffrey W Coombs,2018	197	413	0.48	[0.43; 0.52]	0.3%
Zahraa Nafeh Aziz,2025	4	18	0.22	[0.06; 0.48]	0.3%
DAVID E. PERLADA,1997	20	46	0.43	[0.29; 0.59]	0.3%
Saleem Mohamedali,2017	2	13	0.15	[0.02; 0.45]	0.3%
Ayumi Fujii,2024	1	41	0.02	[0.00; 0.13]	0.3%
Ying−Chi Huang,2024	404	1113	0.36	[0.33; 0.39]	0.3%
S. Li,2006	1	86	0.01	[0.00; 0.06]	0.3%
Soo Tein Ngoi,2021	2	22	0.09	[0.01; 0.29]	0.3%
SÜKRAN SABA ÇOPUR,2016	89	93	0.96	[0.89; 0.99]	0.3%
Nibedita Haldar,2017	9	70	0.13	[0.06; 0.23]	0.3%
Ali Jahansepas,2019	0	8	0.00	[0.00; 0.37]	0.3%
Kyungmin Huh,2023	59	241	0.24	[0.19; 0.30]	0.3%
Yaeghob Sharifi,2012	35	68	0.51	[0.39; 0.64]	0.3%
Ahmad Farajzadeh Sheikh,2019	24	73	0.33	[0.22; 0.45]	0.3%
G. S. Simonsen,2002	0	82	0.00	[0.00; 0.04]	0.3%
D Landman,1999	61	69	0.88	[0.78; 0.95]	0.3%
**Random effects model**		**94793**	**0.25**	**[0.21; 0.29]**	**100.0%**

Heterogeneity: $I^2 = 99.3\%$, $\tau^2 = 0.1517$, $p = 0$
