# Supplementary material for: Global Prevalence of Vancomycin‐Resistant Enterococcus faecium and Enterococcus faecalis Isolated From Clinical and Environmental Sources: A Systematic Review and Meta‐Analysis
Source: Microbiologyopen. 2026 Oct 13;15(5):e70434. doi: 10.1002/mbo3.70434 (PMC13662538; doi:10.1002/mbo3.70434)

Study	Events	Total	Proportion	95%−CI	Weight
Y−J. Kim,2018	0	30	0.00	[0.00; 0.12]	0.3%
Heike von Baum,1999	7	188	0.04	[0.02; 0.08]	0.3%
John C. Christenson,1998	0	13	0.00	[0.00; 0.25]	0.2%
Mariarosaria Boccella,2021	48	2660	0.02	[0.01; 0.02]	0.3%
Mohan Bilikallahalli Sannathimmappa,2023	0	91	0.00	[0.00; 0.04]	0.3%
Dan Alexandru Toc,2023	9	54	0.17	[0.08; 0.29]	0.3%
Maniya Arshadi,2019	4	55	0.07	[0.02; 0.18]	0.3%
Zahraa Nafeh Aziz,2025	9	63	0.14	[0.07; 0.25]	0.3%
Janet M. Manson,2004	18	152	0.12	[0.07; 0.18]	0.3%
Anna Woz´niak−Biel,2019	6	41	0.15	[0.06; 0.29]	0.3%
Farhana Binte Ferdous,2023	23	44	0.52	[0.37; 0.68]	0.3%
Francesca Paola Nocera,2021	7	15	0.47	[0.21; 0.73]	0.3%
Saba Asgharzadeh,2021	0	80	0.00	[0.00; 0.05]	0.3%
Sung Young Lee,2024	11	1227	0.01	[0.00; 0.02]	0.3%
Adam,2011	1	360	0.00	[0.00; 0.02]	0.3%
Sarika Jain,2011	0	36	0.00	[0.00; 0.10]	0.3%
Kaur,2015	1	4	0.25	[0.01; 0.81]	0.2%
Billington,2014	12	169	0.07	[0.04; 0.12]	0.3%
Armin,2017	7	9	0.78	[0.40; 0.97]	0.2%
Geoffrey W. Coombs,2014	3	658	0.00	[0.00; 0.01]	0.3%
Fatima Sana,2019	0	7	0.00	[0.00; 0.41]	0.2%
Youn Jeong Kima,2019	0	17	0.00	[0.00; 0.20]	0.3%
Coombs,GW,2014	4	658	0.01	[0.00; 0.02]	0.3%
Geoffrey W Coombs,2014	1	463	0.00	[0.00; 0.01]	0.3%
Geoffrey W Coombs,2016	1	523	0.00	[0.00; 0.01]	0.3%
Geoffrey W Coombs,2018	2	562	0.00	[0.00; 0.01]	0.3%
Geoffrey W Coombs,2018	2	595	0.00	[0.00; 0.01]	0.3%
Geoffrey W Coombs,2020	0	676	0.00	[0.00; 0.01]	0.3%
Geoffrey W Coombs,2020	1	699	0.00	[0.00; 0.01]	0.3%
Jing−xian Yang,2024	2	72	0.03	[0.00; 0.10]	0.3%
Geoffrey W Coombs,2023	0	812	0.00	[0.00; 0.00]	0.3%
Vanja Piezzi,2020	1	3196	0.00	[0.00; 0.00]	0.3%
Zrinka Todoric,2022	7	256	0.03	[0.01; 0.06]	0.3%
Gümüş,2023	7	216	0.03	[0.01; 0.07]	0.3%
M. Falcone,2019	0	28	0.00	[0.00; 0.12]	0.3%
Anita Williams,2024	0	122	0.00	[0.00; 0.03]	0.3%
Geoffrey W Coombs,2024	0	828	0.00	[0.00; 0.00]	0.3%
Jesús Fortún,2002	0	522	0.00	[0.00; 0.01]	0.3%
Ad C. Fluit,2000	5	411	0.01	[0.00; 0.03]	0.3%
Kapoor,2005	0	10	0.00	[0.00; 0.31]	0.2%
Assadian,2002	1	22	0.05	[0.00; 0.23]	0.3%
Giuseppe Todeschini−Cristina,2005	2	52	0.04	[0.00; 0.13]	0.3%
DAVID E. PERLADA,1997	0	108	0.00	[0.00; 0.03]	0.3%
Gary A. Noskin,1995	0	56	0.00	[0.00; 0.06]	0.3%
Tariq AA Madani,1999	0	116	0.00	[0.00; 0.03]	0.3%
Jae−Cheol Kwon,2012	0	15	0.00	[0.00; 0.22]	0.3%
J. Gray,1994	1	46	0.02	[0.00; 0.12]	0.3%
K. Gopalkrishna Bhat,1997	0	35	0.00	[0.00; 0.10]	0.3%
Maria Cecilia Di Pentima,2014	3	107	0.03	[0.01; 0.08]	0.3%
Sung−Yeon Cho,2013	0	23	0.00	[0.00; 0.15]	0.3%
Hyeah Choi,2022	0	12	0.00	[0.00; 0.26]	0.2%
M. Kierzkowska,2014	0	6	0.00	[0.00; 0.46]	0.2%
M. L. FERNA´ NDEZ−GUERRERO,2002	0	74	0.00	[0.00; 0.05]	0.3%
Noah Hackney,2019	2	22	0.09	[0.01; 0.29]	0.3%
Daniel Hornuss,2024	3	1229	0.00	[0.00; 0.01]	0.3%
Derek F. J. Brown,2008	25	875	0.03	[0.02; 0.04]	0.3%
Lucia Mališová,2021	0	651	0.00	[0.00; 0.01]	0.3%
Chandrabhan Rao,2020	0	134	0.00	[0.00; 0.03]	0.3%
Kuldeep Yadav,2023	29	109	0.27	[0.19; 0.36]	0.3%
David P. Kateete,2019	0	72	0.00	[0.00; 0.05]	0.3%
Jann−Tay Wang,2013	1	1696	0.00	[0.00; 0.01]	0.3%
Francis F. Arhin,2009	87	1738	0.05	[0.04; 0.06]	0.3%
S. Li,2006	4	234	0.02	[0.00; 0.04]	0.3%
Garima Yadav,2017	14	169	0.08	[0.05; 0.14]	0.3%
N.N.I. Mastor,2021	1	34	0.03	[0.00; 0.15]	0.3%
Nidhi Sood,2024	1	12	0.08	[0.00; 0.38]	0.2%
Tsering Yangzom,2020	6	125	0.05	[0.02; 0.10]	0.3%
Hanane Zerrouki,2021	15	65	0.23	[0.14; 0.35]	0.3%
Kaur,2015	0	11	0.00	[0.00; 0.28]	0.2%
Ramonalseppi,2020	0	24	0.00	[0.00; 0.14]	0.3%
Acácio Salamandane,2023	7	8	0.88	[0.47; 1.00]	0.2%
A. BELICOVA,2007	0	49	0.00	[0.00; 0.07]	0.3%
Mehmet Elmali,2018	2	13	0.15	[0.02; 0.45]	0.2%
Onaran,2019	14	16	0.88	[0.62; 0.98]	0.3%
Aylin Kasimoglu−Dogru,2010	0	16	0.00	[0.00; 0.21]	0.3%
R.N. Gruneberg,1998	13	1237	0.01	[0.01; 0.02]	0.3%
Toshiki Kajihara,2015	26	144	0.18	[0.12; 0.25]	0.3%
Birgul Kacmaz,2005	0	207	0.00	[0.00; 0.02]	0.3%
G. NICOLETTI,2006	0	260	0.00	[0.00; 0.01]	0.3%
Helio S. Sader,2011	195	4312	0.05	[0.04; 0.05]	0.3%
Saraswathy Palanisamy,2013	0	98	0.00	[0.00; 0.04]	0.3%
Somayyeh Saeidi,2017	5	124	0.04	[0.01; 0.09]	0.3%
Nagham Joudeh,2023	1	9	0.11	[0.00; 0.48]	0.2%
Gülsen HAZIROLAN,2020	68	336	0.20	[0.16; 0.25]	0.3%
PO−REN HSUEH,2001	0	80	0.00	[0.00; 0.05]	0.3%
N. KOBAYASHI,2000	0	226	0.00	[0.00; 0.02]	0.3%
YUTAKA NISHIMOTO,2005	0	224	0.00	[0.00; 0.02]	0.3%
Y. HIRAKATA,1997	0	49	0.00	[0.00; 0.07]	0.3%
Franz−Josef Schmitz,1999	0	403	0.00	[0.00; 0.01]	0.3%
M.G. Karmarkar,2004	1	10	0.10	[0.00; 0.45]	0.2%
C. J. Henwood,2000	4	875	0.00	[0.00; 0.01]	0.3%
RIKKE LYKKE POULSEN,1999	0	49	0.00	[0.00; 0.07]	0.3%
P A Mackowiak,1989	0	329	0.00	[0.00; 0.01]	0.3%
Priyanka Paul Biswas,2013	3	55	0.05	[0.01; 0.15]	0.3%
Malik,2022	6	52	0.12	[0.04; 0.23]	0.3%
Beata Dolka,2020	2	17	0.12	[0.01; 0.36]	0.3%
Aslantas,2019	0	295	0.00	[0.00; 0.01]	0.3%
Ümmühan Çay,2022	0	11	0.00	[0.00; 0.28]	0.2%
Se−Hyung Kim,2020	0	9	0.00	[0.00; 0.34]	0.2%
Vittoria Cinquepalmi,2012	0	1	0.00	[0.00; 0.97]	0.1%
Ramonalseppi,2020	4	20	0.20	[0.06; 0.44]	0.3%
Teresa M. Braga,2012	7	53	0.13	[0.05; 0.25]	0.3%
Daniel,2017	10	100	0.10	[0.05; 0.18]	0.3%
Erick Odoyo,2023	0	1	0.00	[0.00; 0.97]	0.1%
Raoudha Dziri,2016	0	8	0.00	[0.00; 0.37]	0.2%
Silvia Monteiro,2020	6	20	0.30	[0.12; 0.54]	0.3%
Houssem Ben Yahia,2018	3	53	0.06	[0.01; 0.16]	0.3%
Ellie Hershberger,2005	0	656	0.00	[0.00; 0.01]	0.3%
Iseppi,2015	0	36	0.00	[0.00; 0.10]	0.3%
S.A. Ali,2013	8	27	0.30	[0.14; 0.50]	0.3%
Erki Aun,2021	2	61	0.03	[0.00; 0.11]	0.3%
Jaafar Rezvani,2016	6	91	0.07	[0.02; 0.14]	0.3%
Daniel,2017	12	120	0.10	[0.05; 0.17]	0.3%
Kamonnaree Chotinantakul,2017	0	82	0.00	[0.00; 0.04]	0.3%
Bruna C. Gomes,2008	0	80	0.00	[0.00; 0.05]	0.3%
Nam Hee Kim,2020	0	202	0.00	[0.00; 0.02]	0.3%
Diana Brlek Gorski,2024	1	30	0.03	[0.00; 0.17]	0.3%
S. Düztaslar,2024	2	28	0.07	[0.01; 0.24]	0.3%
G. Pesavento,2014	2	114	0.02	[0.00; 0.06]	0.3%
Lynette M. Johnston,2004	0	38	0.00	[0.00; 0.09]	0.3%
Min−Chan Kim,2017	1	204	0.00	[0.00; 0.03]	0.3%
Malihe Talebi,2008	0	72	0.00	[0.00; 0.05]	0.3%
Athraa Harjan Mohsen Alduhaidhawi,2022	15	50	0.30	[0.18; 0.45]	0.3%
Elham Jannatia,2023	1	33	0.03	[0.00; 0.16]	0.3%
Luca Busani,2004	6	106	0.06	[0.02; 0.12]	0.3%
Bhatt,2015	6	150	0.04	[0.01; 0.09]	0.3%
Anna Woz´niak−Biel,2019	8	45	0.18	[0.08; 0.32]	0.3%
Barbara Araújo dos Santos,2020	32	95	0.34	[0.24; 0.44]	0.3%
Robert S. Dungan,2021	0	37	0.00	[0.00; 0.09]	0.3%
Grebstad Rabbi Amuasi−Felicia A. Owusu,2023	0	96	0.00	[0.00; 0.04]	0.3%
Luca Busani,2004	15	48	0.31	[0.19; 0.46]	0.3%
David Patrick Kateete,2013	3	3	1.00	[0.29; 1.00]	0.2%
Herbert Tomaso−Heinrich,2021	13	17	0.76	[0.50; 0.93]	0.3%
Hanna Rózanska,2019	0	360	0.00	[0.00; 0.01]	0.3%
Aziz Bouymajane,2018	0	11	0.00	[0.00; 0.28]	0.2%
Esra Seker,2023	5	40	0.12	[0.04; 0.27]	0.3%
Hyeon−Jin Kim,2022	0	90	0.00	[0.00; 0.04]	0.3%
Mona A. El−Zamkan,2021	16	20	0.80	[0.56; 0.94]	0.3%
Tiziana Silvetti,2019	0	40	0.00	[0.00; 0.09]	0.3%
Muhammad Arfat Yameen,2013	5	40	0.12	[0.04; 0.27]	0.3%
David Patrick Kateete,2013	0	2	0.00	[0.00; 0.84]	0.1%
Kouidhi,2011	0	17	0.00	[0.00; 0.20]	0.3%
Randa Salah,2008	0	159	0.00	[0.00; 0.02]	0.3%
Manuela Oliveira,2016	0	68	0.00	[0.00; 0.05]	0.3%
Yean Yean Chan,2007	1	47	0.02	[0.00; 0.11]	0.3%
Josep Garcia−Llorens,2025	0	134	0.00	[0.00; 0.03]	0.3%
Vishal C. Shete,2019	3	52	0.06	[0.01; 0.16]	0.3%
Trapti Sharma,2024	7	68	0.10	[0.04; 0.20]	0.3%
Trapti Sharma,2024	7	68	0.10	[0.04; 0.20]	0.3%
Debasmita Dubey,2015	159	265	0.60	[0.54; 0.66]	0.3%
Sachin M Darji,2024	1	80	0.01	[0.00; 0.07]	0.3%
Etinosa O. Igbinosa,2019	8	17	0.47	[0.23; 0.72]	0.3%
Papadimitriou−Olivgeris,2016	12	12	1.00	[0.74; 1.00]	0.2%
Behice Kurtaran, MD,2011	0	25	0.00	[0.00; 0.14]	0.3%
M. Dan,1999	3	37	0.08	[0.02; 0.22]	0.3%
Ricardo Titze,2004	0	70	0.00	[0.00; 0.05]	0.3%
Maureen Boost,2004	0	77	0.00	[0.00; 0.05]	0.3%
Maria Cristina Ossiprandi,2006	0	65	0.00	[0.00; 0.06]	0.3%
Donado−Godoy,2015	6	161	0.04	[0.01; 0.08]	0.3%
Arata Hidano,2015	1	113	0.01	[0.00; 0.05]	0.3%
Veronika Oravcova,2013	5	5	1.00	[0.48; 1.00]	0.2%
Neda Noroozi,2022	6	56	0.11	[0.04; 0.22]	0.3%
Nabil Ben Omar,2023	0	14	0.00	[0.00; 0.23]	0.3%
Birri,2012	1	15	0.07	[0.00; 0.32]	0.3%
Ramonalseppi,2020	0	12	0.00	[0.00; 0.26]	0.2%
Susana Correia,2013	1	1	1.00	[0.02; 1.00]	0.1%
Solayide A. Adesida,2017	3	29	0.10	[0.02; 0.27]	0.3%
Techilo Habtemariam Mengesha,2024	0	65	0.00	[0.00; 0.06]	0.3%
Jannati,2020	0	56	0.00	[0.00; 0.06]	0.3%
Ngbede,2023	0	41	0.00	[0.00; 0.09]	0.3%
SR Moaddab,2004	0	90	0.00	[0.00; 0.04]	0.3%
Morris,1995	11	11	1.00	[0.72; 1.00]	0.2%
Freitas,2006	9	9	1.00	[0.66; 1.00]	0.2%
Jozef Kuzma,2022	9	9	1.00	[0.66; 1.00]	0.2%
Imane Hannaoui,2016	8	45	0.18	[0.08; 0.32]	0.3%
Katarzyna Grudlewska,2023	7	121	0.06	[0.02; 0.12]	0.3%
Kere Kobayashi,2000	0	51	0.00	[0.00; 0.07]	0.3%
Jinglu Sun,2009	0	108	0.00	[0.00; 0.03]	0.3%
Thais de Campos,2015	0	218	0.00	[0.00; 0.02]	0.3%
Ali Jahansepas,2019	0	40	0.00	[0.00; 0.09]	0.3%
NASSIMA DJAHMI,2012	0	80	0.00	[0.00; 0.05]	0.3%
Gülsah Tollu,2020	0	40	0.00	[0.00; 0.09]	0.3%
Félix Carrasco Calzada,2023	3	51	0.06	[0.01; 0.16]	0.3%
Pan,2024	3	1224	0.00	[0.00; 0.01]	0.3%
Fakhri Haghi,2019	0	69	0.00	[0.00; 0.05]	0.3%
Tiffany R. Keepers,2017	5	54	0.09	[0.03; 0.20]	0.3%
Abdelkareem,2016	2	57	0.04	[0.00; 0.12]	0.3%
Nagen Kumar Debata,2013	31	115	0.27	[0.19; 0.36]	0.3%
Samiah Hamad S Al−Mijalli,2022	0	2	0.00	[0.00; 0.84]	0.1%
Mohua Bose,2015	4	115	0.03	[0.01; 0.09]	0.3%
Varun Goel,2016	8	61	0.13	[0.06; 0.24]	0.3%
Kaina Bhonsle,2023	3	36	0.08	[0.02; 0.22]	0.3%
Ayan Kumar,2022	16	140	0.11	[0.07; 0.18]	0.3%
Mahnaz Saifi,2007	13	496	0.03	[0.01; 0.04]	0.3%
Kumar Das,2020	14	81	0.17	[0.10; 0.27]	0.3%
Hesam Alizade,2022	6	50	0.12	[0.05; 0.24]	0.3%
Kiruthiga,2019	0	70	0.00	[0.00; 0.05]	0.3%
Smeeta Huidrom,2016	14	102	0.14	[0.08; 0.22]	0.3%
Mehmet Butcu,2011	1	67	0.01	[0.00; 0.08]	0.3%
Rand R. Hafidh,2023	6	17	0.35	[0.14; 0.62]	0.3%
Raad Saad Luty,2020	8	19	0.42	[0.20; 0.67]	0.3%
Sanjeev Das,2021	18	381	0.05	[0.03; 0.07]	0.3%
Naidoo,2023	5	372	0.01	[0.00; 0.03]	0.3%
Silvi Bozo,2025	31	57	0.54	[0.41; 0.68]	0.3%
Ayan Kumar,2022	14	94	0.15	[0.08; 0.24]	0.3%
Zuzanna Kraszewska,2022	5	389	0.01	[0.00; 0.03]	0.3%
Snežana Brkic,2017	0	5060	0.00	[0.00; 0.00]	0.3%
Félix Carrasco Calzada,2023	1	18	0.06	[0.00; 0.27]	0.3%
Alina Maria Borcan,2024	9	427	0.02	[0.01; 0.04]	0.3%
BAHRAM FATHOLAHZADEH,2006	3	68	0.04	[0.01; 0.12]	0.3%
Qing−Yong Wang,2015	12	252	0.05	[0.02; 0.08]	0.3%
Heba A. Ahmed,2023	38	43	0.88	[0.75; 0.96]	0.3%
Eman A. El−Masry,2015	14	22	0.64	[0.41; 0.83]	0.3%
Maria Bitsori,2005	0	13	0.00	[0.00; 0.25]	0.2%
F. MOHAMMADI,2015	27	128	0.21	[0.14; 0.29]	0.3%
WAVARE SANJAY M,2015	0	112	0.00	[0.00; 0.03]	0.3%
Alina Maria Borcan,2024	9	427	0.02	[0.01; 0.04]	0.3%
Wanqing Zhou,2020	1	1870	0.00	[0.00; 0.00]	0.3%
Ayumi Fujii,2024	0	80	0.00	[0.00; 0.05]	0.3%
Shahram Shahraki,2017	6	63	0.10	[0.04; 0.20]	0.3%
A Tripathi,2016	72	1067	0.07	[0.05; 0.08]	0.3%
Yaeghob Sharifi,2012	10	152	0.07	[0.03; 0.12]	0.3%
Ali Jahansepas,2019	9	125	0.07	[0.03; 0.13]	0.3%
Luciana Furlaneto−Maia,2014	3	7	0.43	[0.10; 0.82]	0.2%
G. S. Simonsen,2002	0	420	0.00	[0.00; 0.01]	0.3%
Mallika Sengupta,2023	14	239	0.06	[0.03; 0.10]	0.3%
Obaid Ullah,2015	32	52	0.62	[0.47; 0.75]	0.3%
Meharunisa Indikar,2024	4	60	0.07	[0.02; 0.16]	0.3%
Chimanjita Phukan,2016	9	54	0.17	[0.08; 0.29]	0.3%
A. Japoni,2009	65	180	0.36	[0.29; 0.44]	0.3%
Narges Dadfarmaa,2013	0	90	0.00	[0.00; 0.04]	0.3%
S. YASLIANI,2009	5	160	0.03	[0.01; 0.07]	0.3%
Fatemeh Shahi,2020	0	17	0.00	[0.00; 0.20]	0.3%
Heba Shehta Said,2019	1	71	0.01	[0.00; 0.08]	0.3%
STEVEN GORDON,1992	2	632	0.00	[0.00; 0.01]	0.3%
Bahram Nikmanesh,2024	13	103	0.13	[0.07; 0.21]	0.3%
Ujjala Ghoshal,2006	0	456	0.00	[0.00; 0.01]	0.3%
Zahraa Nafeh Aziz,2025	9	63	0.14	[0.07; 0.25]	0.3%
Mahmoud M. Tawfick,2020	0	15	0.00	[0.00; 0.22]	0.3%
Ying−Chi Huang,2024	6	2736	0.00	[0.00; 0.00]	0.3%
Prakaithip Thongkoom,2012	2	4827	0.00	[0.00; 0.00]	0.3%
Nibedita Haldar,2017	15	200	0.07	[0.04; 0.12]	0.3%
Saleem Mohamedali,2017	0	15	0.00	[0.00; 0.22]	0.3%
Shuan Tao,2022	4	62	0.06	[0.02; 0.16]	0.3%
Davood Yadegarynia,2016	8	41	0.20	[0.09; 0.35]	0.3%
Sangjukta Roy,2025	1	135	0.01	[0.00; 0.04]	0.3%
Ghazal Zolfgar,2024	24	82	0.29	[0.20; 0.40]	0.3%
Ahmad Farajzadeh Sheikh,2019	1	15	0.07	[0.00; 0.32]	0.3%
Hossein Samadi Kafil,2013	18	114	0.16	[0.10; 0.24]	0.3%
Geoffrey W Coombs,2013	6	1201	0.00	[0.00; 0.01]	0.3%
Masoud Alebouyeh,2005	1	114	0.01	[0.00; 0.05]	0.3%
SÜKRAN SABA ÇOPUR,2016	4	23	0.17	[0.05; 0.39]	0.3%
Xiomara Pe´rez−Herna´ ndez,2002	1	377	0.00	[0.00; 0.01]	0.3%
Leila Ben Said,2015	0	4	0.00	[0.00; 0.60]	0.2%
E. Seker,2023	10	31	0.32	[0.17; 0.51]	0.3%
Diana Brlek Gorski,2024	7	30	0.23	[0.10; 0.42]	0.3%
SUMRU ÇITAK,2004	60	62	0.97	[0.89; 1.00]	0.3%
P. Poeta,2005	0	73	0.00	[0.00; 0.05]	0.3%
Javier Aznar,2012	23	2068	0.01	[0.01; 0.02]	0.3%
Stevens Kisaka,2022	0	19	0.00	[0.00; 0.18]	0.3%
Hamid Heidari,2016	0	46	0.00	[0.00; 0.08]	0.3%
Ahmed,2014	0	54	0.00	[0.00; 0.07]	0.3%
Natália Conceição,2011	2	282	0.01	[0.00; 0.03]	0.3%
Alina−Viorica Iancu,2023	2	41	0.05	[0.01; 0.17]	0.3%
Nadjette Bourafa−Ce´,2016	0	64	0.00	[0.00; 0.06]	0.3%
Kowalska−Krochmal,2008	0	1312	0.00	[0.00; 0.00]	0.3%
Hanna Billström,2008	14	263	0.05	[0.03; 0.09]	0.3%
Banerjee,2015	9	155	0.06	[0.03; 0.11]	0.3%
Marzieh Aligholi,2008	13	320	0.04	[0.02; 0.07]	0.3%
Michael Brandon,2013	4	1112	0.00	[0.00; 0.01]	0.3%
Douglas J. Biedenbach,2007	1	255	0.00	[0.00; 0.02]	0.3%
Daniel,2017	2	30	0.07	[0.01; 0.22]	0.3%
Keryn J Christiansen,2007	3	1987	0.00	[0.00; 0.00]	0.3%
Andrew J. Denisuik,2018	0	198	0.00	[0.00; 0.02]	0.3%
SULE CELIK,2014	0	63	0.00	[0.00; 0.06]	0.3%
Dehariya,2024	19	154	0.12	[0.08; 0.19]	0.3%
S. Sreeja,2012	0	97	0.00	[0.00; 0.04]	0.3%
Malihe Talebi,2008	0	365	0.00	[0.00; 0.01]	0.3%
Farooq Ahmad,2024	10	61	0.16	[0.08; 0.28]	0.3%
MM Salem−Bekhit,2011	3	166	0.02	[0.00; 0.05]	0.3%
Mengjun Cheng,2017	22	40	0.55	[0.38; 0.71]	0.3%
Aasish Karna,2019	13	42	0.31	[0.18; 0.47]	0.3%
Babak POURAKBARI,2012	15	91	0.16	[0.10; 0.26]	0.3%
Hasan Ejaz,2019	4	139	0.03	[0.01; 0.07]	0.3%
Fawzia E Alotaibi,2017	2	168	0.01	[0.00; 0.04]	0.3%
Lin Huang,2019	151	31709	0.00	[0.00; 0.01]	0.3%
Robert Ducu,2019	12	60	0.20	[0.11; 0.32]	0.3%
Mona NASAJ,2015	9	175	0.05	[0.02; 0.10]	0.3%
Arockia Doss Susai backiam,2023	167	388	0.43	[0.38; 0.48]	0.3%
Fahimeh Hajiahmadi,2016	2	76	0.03	[0.00; 0.09]	0.3%
Azin Sattari−Maraji,2019	6	67	0.09	[0.03; 0.18]	0.3%
Jéssica Lopes−Teresa Conceição,2024	0	138	0.00	[0.00; 0.03]	0.3%
Wei Jia,2014	0	382	0.00	[0.00; 0.01]	0.3%
Lo,2011	20	376	0.05	[0.03; 0.08]	0.3%
Ali Jahansepas,2017	9	125	0.07	[0.03; 0.13]	0.3%
Ashfaq Ahmad Shah,2022	7	83	0.08	[0.03; 0.17]	0.3%
Katharine Bar,2006	0	30	0.00	[0.00; 0.12]	0.3%
Abdulhakim Abamecha,2015	0	34	0.00	[0.00; 0.10]	0.3%
Lei Ge, MD,2022	0	11	0.00	[0.00; 0.28]	0.2%
Nuhamin Melaku Gebremariam,2022	1	24	0.04	[0.00; 0.21]	0.3%
Ali Al Bshabshe,2024	15	561	0.03	[0.02; 0.04]	0.3%
Sanja Jakovac,2016	12	29	0.41	[0.24; 0.61]	0.3%
Soo Tein Ngoi,2021	4	23	0.17	[0.05; 0.39]	0.3%
Hota,2024	5	75	0.07	[0.02; 0.15]	0.3%
Wenwei Ouyang,2016	0	32	0.00	[0.00; 0.11]	0.3%
Natkamon Saenhom,2022	5	14	0.36	[0.13; 0.65]	0.3%
Christoph Lu¨bbert,2015	18	236	0.08	[0.05; 0.12]	0.3%
M. Kresken,2009	0	149	0.00	[0.00; 0.02]	0.3%
Dowzicky,2019	0	215	0.00	[0.00; 0.02]	0.3%
I. Gawryszewska,2016	0	140	0.00	[0.00; 0.03]	0.3%
Bogut,2021	0	17	0.00	[0.00; 0.20]	0.3%
Diana Brlek Gorski,2024	3	29	0.10	[0.02; 0.27]	0.3%
Hitkova,2019	0	4	0.00	[0.00; 0.60]	0.2%
Abat,2015	0	2507	0.00	[0.00; 0.00]	0.3%
M. Pinholt,2013	7	457	0.02	[0.01; 0.03]	0.3%
Malka,2023	18	744	0.02	[0.01; 0.04]	0.3%
Xiaoyan Liao,2024	0	58	0.00	[0.00; 0.06]	0.3%
Ernesto Calderón−Jaimes,2003	0	60	0.00	[0.00; 0.06]	0.3%
M.T. Akhi,2009	1	124	0.01	[0.00; 0.04]	0.3%
Carlos Bantar,2000	0	305	0.00	[0.00; 0.01]	0.3%
Roberta Fontana,1998	100	9169	0.01	[0.01; 0.01]	0.3%
Kyungwon Lee−Sook−Jin Jang,2003	101	10076	0.01	[0.01; 0.01]	0.3%
JAN M. BELL,1998	16	60	0.27	[0.16; 0.40]	0.3%
Bell,2003	0	51	0.00	[0.00; 0.07]	0.3%
Bell,2003	0	89	0.00	[0.00; 0.04]	0.3%
Bell,2003	0	70	0.00	[0.00; 0.05]	0.3%
Bell,2003	0	49	0.00	[0.00; 0.07]	0.3%
Bell,2003	0	72	0.00	[0.00; 0.05]	0.3%
Bell,2003	0	150	0.00	[0.00; 0.02]	0.3%
Markus Hufnagel,2007	0	8	0.00	[0.00; 0.37]	0.2%
Edet E. Udo,2002	6	354	0.02	[0.01; 0.04]	0.3%
Mona Abdel Monem Esmail,2019	14	26	0.54	[0.33; 0.73]	0.3%
Folasade Muibat Adeyemi,2021	24	24	1.00	[0.86; 1.00]	0.3%
CARINA CLAESSON,2008	0	429	0.00	[0.00; 0.01]	0.3%
L. Adhikari,2016	26	82	0.32	[0.22; 0.43]	0.3%
Linda Labecka,2024	6	82	0.07	[0.03; 0.15]	0.3%
CARINA CLAESSON,2007	1	508	0.00	[0.00; 0.01]	0.3%
W. Grzybowska,2009	12	12	1.00	[0.74; 1.00]	0.2%
Mei−Chun Lee,2021	47	5738	0.01	[0.01; 0.01]	0.3%
Min Liu,2023	0	9	0.00	[0.00; 0.34]	0.2%
Dilek BULUT,2024	7	290	0.02	[0.01; 0.05]	0.3%
Sarah V. Walker,2020	0	853	0.00	[0.00; 0.00]	0.3%
D Landman,1999	26	430	0.06	[0.04; 0.09]	0.3%
**Random effects model**		**144493**	**0.05**	**[0.03; 0.06]**	**100.0%**

Heterogeneity: $I^2 = 95.6\%$, $\tau^2 = 0.0662$, $p = 0$

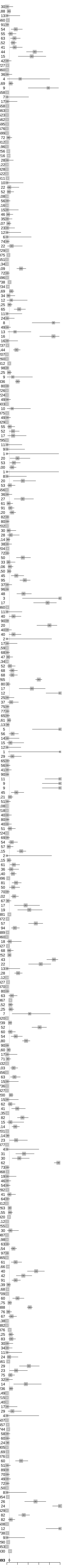

Supplement: Supplementary file 2 [file MBO3-15-e70434-s003.pdf]
