# Supplementary material for: Global Prevalence of Vancomycin‐Resistant Enterococcus faecium and Enterococcus faecalis Isolated From Clinical and Environmental Sources: A Systematic Review and Meta‐Analysis
Source: Microbiologyopen. 2026 Oct 13;15(5):e70434. doi: 10.1002/mbo3.70434 (PMC13662538; doi:10.1002/mbo3.70434)
Supplement: Supplementary file 3 [file MBO3-15-e70434-s005.docx]

((("Prevalence” ) OR ( "frequency") OR (“Epidemiology”) OR (“Rate”) OR (“incidence”)) AND ((Enteroccoci*) OR (“Enterococcus faecalis”) OR (“Enterococcus faecium”) OR (“E. faecalis”) OR (“E. faecium”) OR ("Streptococcus Group D") OR ("Streptococcus faecalis") OR ("Streptococcus faecium") OR ("Micrococcus zymogenes") OR ("streptococcus fecalis") OR ("Streptococcus liquefaciens") OR ("Paraghurt")) AND ((“Vancomycin resistan*”) OR (Vancomycin) OR (GRE) OR (“glycopeptide-resistan*”) OR (Van) OR (VRE)))

PUBMED SEARCH:

26 April 2025

Result: 4303

("Prevalence"[Mh] OR Prevalence[TIAB] OR "Period Prevalence"[TIAB] OR "Point Prevalence"[TIAB] OR "prevalence study"[TIAB] OR "frequency"[TIAB]) AND ("Vancomycin-Resistan* Enterococci"[Mh] OR "Enterococcus"[Mh] OR "Enterococcus faecalis"[Mh] OR "Enterococcus faecium"[Mh] OR "Vancomycin Resistan* Enterococci"[TIAB] OR VRE[TIAB] OR "Vancomycin resistan* Enterococcus faecalis"[TIAB] OR "vancomycin resistan* Enterococcus faecium"[TIAB] OR Enterococcus [TIAB] OR "Enterococcus faecalis"[TIAB] OR "Enterococcus faecium"[TIAB] OR "E. faecalis"[TIAB] OR "Streptococcus Group D"[TIAB] OR "Streptococcus faecalis"[TIAB] OR "E. faecium"[TIAB] OR "Streptococcus faecium"[TIAB] OR "VRE (enterococcus) "[TIAB] OR "Micrococcus zymogenes"[TIAB] OR "streptococcus fecalis"[TIAB] OR "Streptococcus liquefaciens"[TIAB] OR "Paraghurt"[TIAB])

EMBASE SEARCH:

26 April 2025

Result: 4,258

('prevalence':ti,ab OR 'frequency':ti,ab OR 'epidemiology':ti,ab OR 'rate':ti,ab OR 'incidence':ti,ab) AND ('enteroccoci*':ti,ab OR 'enterococcus faecalis':ti,ab OR 'enterococcus faecium':ti,ab OR 'e. faecalis':ti,ab OR 'e. faecium':ti,ab OR 'streptococcus group d':ti,ab OR 'streptococcus faecalis':ti,ab OR 'streptococcus faecium':ti,ab OR 'micrococcus zymogenes':ti,ab OR 'streptococcus fecalis':ti,ab OR 'streptococcus liquefaciens':ti,ab OR 'paraghurt':ti,ab) AND ('vancomycin resistan*':ti,ab OR vancomycin:ti,ab OR gre:ti,ab OR 'glycopeptide-resistan*':ti,ab OR 'antibiotic resistan*':ti,ab OR 'antimicrobial resistan*':ti,ab OR 'drug resistan*':ti,ab OR vre:ti,ab OR 'antibiotic':ti,ab OR 'susceptib*':ti,ab)

WOS SEARCH

26 April 2025

Result: 3075

“prevalence study” OR “prevalence” OR “Period Prevalence” OR “Point Prevalence” OR “frequency”

“Vancomycin Resistant Enterococci” OR “VRE” OR “Vancomycin resistance Enterococcus faecalis” OR “vancomycin resistance Enterococcus faecium “ OR “Enterococcus“ OR “Enterococcus faecalis” OR “Enterococcus faecium” OR “E. faecalis” OR “Streptococcus Group D” OR “Streptococcus faecalis” OR “E. faecium” OR “Streptococcus faecium” OR “VAN resistant Enterococcus” OR “VAN-R enterococci” OR “VAN-R Enterococcus” OR “VRE (enterococcus)” OR “vancomycin resistant Enterococcus” OR “Micrococcus zymogenes” OR “streptococcus fecalis” OR “Streptococcus liquefaciens” OR “Paraghurt”

SCOPUS SEARCH:

26 April 2025

Result: 5356

TITLE-ABS-KEY(“prevalence study” OR “prevalence” OR “Period Prevalence” OR “Point Prevalence” OR “frequency”)

TITLE-ABS-KEY(“Vancomycin Resistant Enterococci” OR “VRE” OR “Vancomycin resistance Enterococcus faecalis” OR “vancomycin resistance Enterococcus faecium “ OR “Enterococcus“ OR “Enterococcus faecalis” OR “Enterococcus faecium” OR “E. faecalis” OR “Streptococcus Group D” OR “Streptococcus faecalis” OR “E. faecium” OR “Streptococcus faecium” OR “VRE (enterococcus)” OR “vancomycin resistant Enterococcus” OR “Micrococcus zymogenes” OR “streptococcus fecalis” OR “Streptococcus liquefaciens” OR “Paraghurt”)
